# How Endpoint Definitions Shape Epidemiologic Inference on Tuberculosis Treatment Outcomes: A Secondary Analysis from Lima, Peru

**DOI:** 10.3390/idr18050108

**Published:** 2026-09-26

**Authors:** Víctor Juan Vera-Ponce, Jhosmer Ballena-Caicedo, Namibia Jherly Cervera Vasquez, Marcos Daniel García-Rodríguez, Jorge Alejandro Yumpo Ynga, Fiorella E. Zuzunaga-Montoya

**Affiliations:** Facultad de Medicina (FAMED), Universidad Nacional Toribio Rodríguez de Mendoza de Amazonas (UNTRM), Chachapoyas 01001, Amazonas, Peru; 7330178022@untrm.edu.pe (J.B.-C.); 7679509541@untrm.edu.pe (N.J.C.V.); marcos.garcia@untrm.edu.pe (M.D.G.-R.); 7215802831@untrm.edu.pe (J.A.Y.Y.); fiorella.zuzunaga@untrm.edu.pe (F.E.Z.-M.)

**Keywords:** tuberculosis, loss to follow-up, treatment outcomes, Peru, multinomial logistic regression, public health

## Abstract

Background/Objectives: Tuberculosis treatment outcomes are often dichotomized into heterogeneous composites that may obscure outcome-specific associations. We compared baseline characteristics across mutually exclusive out-comes—loss to follow-up (LTFU), death, and not evaluated/transferred versus documented cure—and examined the composite endpoints secondarily in a prospective cohort of adults with smear-positive pulmonary tuberculosis treated in Lima, Peru, in 2010–2011. Methods: The complete-case cohort included 1233 participants. The primary analysis used ridge-penalized multinomial logistic regression (lambda = 0.16); a mean bias-reduced multinomial model assessed sparse-data sensitivity. Results: Overall, 1016 participants were cured, 127 experienced LTFU, 30 died, and 60 were not evaluated/transferred. Drug use was associated primarily with LTFU (aOR 4.15; 95% CI 2.55–6.75), whereas HIV-positive status showed the largest, although imprecisely estimated, association with death (aOR 32.12; 95% CI 8.95–115.33; six deaths among 22 HIV-positive participants). The bias-reduced estimate was similar (aOR 35.56; 95% CI 9.80–129.08). Multidrug-resistant tuberculosis was associated with several non-cure categories; its association with the combined not evaluated/transferred category was largely driven by participants still in treatment at database closure. Composite endpoints changed the magnitude and interpretation of several associations and did not eliminate sparse outcome–exposure cells. Conclusions: Endpoint definitions therefore materially shaped epidemiologic inference. Dis-aggregated outcomes should be reported alongside composite indicators, and findings from this historical cohort should not be interpreted as contemporary risk estimates for Lima.

## 1. Introduction

Tuberculosis remains a public health priority because of its high disease burden, community transmission, and the need for prolonged treatment to achieve favorable clinical and programmatic outcomes. In 2024, the World Health Organization estimated 10.7 million people with tuberculosis worldwide and 1.23 million deaths attributable to the disease, including 150,000 deaths among people with HIV [1]. During the same period, multidrug-resistant or rifampicin-resistant tuberculosis remained a critical problem: an estimated 390,000 incident cases of MDR/RR-TB occurred, but only about two in five affected individuals accessed treatment [2]. Thus, although tuberculosis is preventable and curable, the usefulness of interventions depends not only on diagnosis and treatment initiation but also on treatment continuity and the correct classification of outcomes.

In the region of the Americas, tuberculosis remains a relevant programmatic problem, and the Pan American Health Organization continues to monitor regional incidence, mortality, case notification, and progress toward End TB Strategy targets [3]. In Peru, the burden of disease remains high and territorially concentrated. In 2024, 32,950 tuberculosis cases were reported, 54.7% of which were concentrated in Lima and Callao [4]. In addition, the National Institute of Health reported 1259 MDR-TB cases, 877 RR-TB cases, and 10 XDR-TB cases in 2024; Lima and Callao accounted for 54.7% of tuberculosis cases, 73.3% of MDR-TB cases, and 90% of XDR-TB cases [5]. This pattern justifies a detailed examination of the treatment outcomes in high-burden urban settings, where outcome categories may reflect distinct clinical, social, and programmatic processes.

The contemporary burden figures provide current programmatic context, whereas the analytic cohort reflects care delivered in 2010–2011. Since then, diagnostic pathways, HIV care, and MDR/RR-TB treatment have changed substantially, including the introduction and expansion of shorter all-oral regimens recommended by the World Health Organization [6]. Accordingly, the present study should be interpreted primarily as a methodological evaluation of endpoint architecture rather than as an estimate of contemporary risk patterns in Lima.

Standardizing antituberculosis treatment outcomes is necessary to compare indicators across programs, periods, and populations. The WHO revised its definitions for tuberculosis and drug-resistant tuberculosis, including categories such as cured, treatment completed, treatment failed, died, loss to follow-up, and not evaluated [7]. These categories are not interchangeable. Loss to follow-up represents documented treatment interruption or non-initiation; death is a definitive clinical outcome; and transfer or absence of a final evaluation reflects programmatic uncertainty about the outcome at the facility of origin. When these categories are grouped in binary form, for example, as successful versus unsuccessful treatment or loss to follow-up versus no loss to follow-up, estimated associations may depend on the outcome architecture selected. Beyond influencing epidemiologic estimates, endpoint architecture also determines which programmatic problem is being monitored and may therefore shape performance evaluations, intervention priorities, and resource allocations.

Previous evidence from a Lima cohort identified characteristics associated with treatment default, now termed loss to follow-up, using a binary model that compared patients with default against all other outcomes [8]. Subsequently, a predictive analysis developed a nomogram to estimate the probability of default under facility-based directly observed therapy using the same dataset and a similar binary outcome [9,10]. These studies established relevant associations and explored individual prediction, but they did not evaluate whether factors associated with loss to follow-up are the same as those associated with death, transfer, absence of documented cure, or other composite outcomes. This distinction is methodologically important because decisions to collapse categories can alter the magnitude, direction, or interpretation of observed associations.

Accordingly, the primary objective was to compare associations between baseline characteristics and the mutually exclusive outcomes of LTFU, death, and not evaluated/transferred by using documented cure as the reference. Secondary analyses evaluated how these associations changed when outcome categories were collapsed into composite binary endpoints. This study was intended to examine the inferential consequences of endpoint definition rather than to develop a new prediction model or reproduce the identification of factors associated with treatment default.

## 2. Materials and Methods

### 2.1. Study Design

We conducted a secondary analysis of a prospective cohort of patients with smear-positive pulmonary tuberculosis treated in a high-incidence area of Lima, Peru. The manuscript was prepared in accordance with the STROBE recommendations for observational cohort studies [11]. The completed STROBE checklist is provided in Table S1.

### 2.2. Data Source

We used publicly available, anonymized data from the Dryad dataset ‘Data from: Patient characteristics associated with tuberculosis treatment default: a cohort study in a high-incidence area of Lima, Peru’ [9].

The public dataset corresponds to the original study by Lackey and colleagues, which included adults with a first episode of smear-positive pulmonary tuberculosis who were enrolled between January 2010 and December 2011, with follow-up of treatment outcomes recorded in health services [8]. The analyzed file was Lima TB Treatment Default Data.xls and contains baseline demographic, socioeconomic, behavioral, and clinical variables, as well as treatment outcomes and time to default for patients who experienced that event.

### 2.3. Population and Sample

The source population comprised patients included in the public dataset. For the primary analysis, records with a documented treatment outcome were considered eligible.

Records without an outcome and those with missing data in the baseline variables required for the complete-case analytic cohort were excluded. This decision made it possible to reproduce the analytic population of the original study and maintain direct comparability with its estimates. Because the proportion of records excluded due to missing covariates was limited, complete-case analysis was considered appropriate for the primary analysis. The influence of missing data was evaluated through sensitivity analyses.

### 2.4. Variables

The original treatment outcome variable included the categories cured, default, Died, transferred out, and still in treatment. In the present manuscript, default was referred to as a loss to follow-up, or LTFU, in accordance with World Health Organization terminology [7,12]. The primary outcome was a four-category nominal variable: documented cure, LTFU, death, and not evaluated/transferred. Transferred out indicates a continuation of care outside the reporting facility with an unknown final outcome, whereas still in treatment indicates an ongoing, nonterminal status at database closure. Neither component was interpreted as treatment failure; their combination was used only to represent the absence of a documented final outcome at the source facility, and the components were examined separately in sensitivity analyses.

Two secondary binary endpoints were analyzed. The first contrasted an absence of a documented cure, excluding ongoing treatment, with a documented cure; this endpoint included LTFU, death, and transfer, while participants still in treatment at database closure were excluded because they had not reached a terminal outcome. The second, strict unfavorable outcome, contrasted LTFU or death with a documented cure and excluded transferred and still-in-treatment records. A broader closure-status definition that additionally classified still-in-treatment records as lacking a documented cure was retained for sensitivity analysis.

The prespecified covariates for the primary model were categorized as age, sex, incomplete secondary education, alcohol use at least once per week, history of drug use, multidrug-resistant tuberculosis, body mass index, or HIV status. Age was analyzed using the categories available in the dataset. Body mass index was analyzed as normal, underweight, or overweight/obesity. HIV status was analyzed as negative, positive, or test not performed. The test-not-performed category was treated as an observed programmatic condition, not as missing data, because it reflects an absence of documented screening. Other baseline variables, including marital status, poverty, history of imprisonment, smoking, rehabilitation, chronic disease, and diabetes, were used for description and extended sensitivity analyses.

### 2.5. Statistical Analysis

Baseline characteristics were described using absolute frequencies and percentages. Significance tests were not performed for the main descriptive table because the objective was to characterize the sample rather than conduct bivariate inference on differences between groups.

The primary analysis used multinomial logistic regression with documented cure as the reference category. This model was selected because treatment outcomes were mutually exclusive and because the objective was to compare, within a single analytic structure, the associations of covariates with LTFU, death, and not evaluated/transferred. Ordinal regression was not used because the outcome categories do not have a single monotonic clinical order. Survival models and competing-risk models were also not used because time to event was recorded only for patients with LTFU and comparable censoring or follow-up times were unavailable for cure, death, transfer, or remaining in treatment.

The primary multinomial model included all prespecified covariates and did not use automatic variable selection. Adjusted odds ratios (aORs) for each outcome category versus cure were estimated with 95% confidence intervals. To stabilize estimation in sparse outcome–exposure cells, we maximized a ridge-penalized multinomial log-likelihood with lambda = 0.16 applied only to the non-intercept coefficients. Lambda was fixed in the analysis script and was not selected according to statistical significance or predictive performance. Optimization used the L-BFGS-B algorithm with a maximum of 180 iterations, ftol = 1 × 10^−10^, gtol = 1 × 10^−6^, and maxls = 50; the model converged after 64 iterations. Wald confidence intervals were calculated using the generalized inverse of the penalized Hessian. Coefficient heterogeneity across outcome categories was evaluated using approximate Wald tests based on the same covariance matrix.

As a sparse-data sensitivity analysis, we fitted a mean bias-reduced baseline-category multinomial logistic regression using the same complete-case cohort, prespecified covariates, and cure reference outcome. Mean bias reduction used the Firth/Jeffreys adjusted-likelihood approach, which yields finite estimates under separation [13,14,15]. Wald confidence intervals were calculated from the expected Fisher information at the bias-reduced estimates. This analysis evaluated coefficient stability and did not replace the prespecified ridge-penalized primary model.

Secondary binary endpoints were analyzed using ridge-penalized logistic models with the same covariate set as in the primary analysis. These models were used to evaluate how estimates changed when heterogeneous outcomes were collapsed into an absence of documented cure, excluding ongoing treatment, strict unfavorable outcomes, and a broader closure-status definition. In addition, two binary contrasts related to the original study were reproduced: LTFU versus all other outcomes, and LTFU versus cure only.

Sensitivity analyses were conducted to evaluate the robustness of the findings. First, unadjusted associations were estimated for each outcome-specific contrast. Second, the primary complete-case model was compared with a complete-case analysis restricted to model covariates and with a single stochastic hot-deck imputation sensitivity analysis among participants with recorded outcomes. The outcome was not imputed. The hot-deck analysis was used only to examine the direction and approximate magnitude of estimates and was not treated as a full multiple-imputation correction for missing-data uncertainties. Third, the not evaluated/transferred category was evaluated using specifications that separated transferred participants from those still in treatment, and the composite endpoint was evaluated after excluding ongoing treatment. Fourth, an extended model including additional available baseline variables was fitted. Finally, standardized marginal probabilities were estimated for selected exposures by averaging individual predictions over the empirical distribution of covariates. All analyses were performed in Python version 3.11.9 (Python Software Foundation, Beaverton, OR, USA) using pandas, NumPy, SciPy, and Matplotlib; the sparse-data sensitivity analysis used a validated implementation of mean bias reduction.

### 2.6. Ethical Considerations

The original cohort protocol was approved by the Institutional Review Board of Universidad Peruana Cayetano Heredia and the East Lima DISA, and all participants provided written informed consent [8]. The present study used only the publicly available, anonymized Dryad dataset. The authors had no access to direct identifiers, did not contact participants, and made no attempt at re-identification. Secondary analyses of anonymized data still require preservation of confidentiality and compliance with applicable institutional requirements [16]. No new participant consent was required for this secondary analysis.

## 3. Results

### 3.1. Analytic Sample

The public file included 1293 records. Treatment outcome was documented in 1278 participants, and the complete-case analytic cohort included 1233 participants after excluding records with an absent outcome or missing data in the required baseline variables. In this analytic cohort, 1016 participants had a documented cure, 127 had LTFU, 30 died, and 60 were classified as not evaluated/transferred; the latter combined category included 40 transferred participants and 20 participants still in treatment. Sample selection is summarized in Figure S1. The revised recoding architecture for the original, nominal, and composite outcomes is shown in Figure 1, and the analytic recoding of the variables is detailed in Table S2. The missing-data pattern was concentrated in a limited number of baseline covariates and is described in Table S3.

### 3.2. Baseline Characteristics

Table 1 presents the baseline characteristics according to the primary outcome category. In the analytic cohort, 743 patients were male, corresponding to 60.3% of the sample. Overall, 514 patients had not completed secondary education, 233 reported alcohol use at least once per week, 190 had a history of drug use, 81 had multidrug-resistant tuberculosis, 151 were underweight, and 295 had no HIV test performed.

Compared with cured patients, patients with LTFU had a higher proportion of male sex, incomplete secondary education, weekly alcohol use, history of drug use, multidrug-resistant tuberculosis, being underweight, and a HIV test was not performed. Among patients with LTFU, 98 of 127 were male, 75 had not completed secondary education, 55 reported weekly alcohol use, 63 had a history of drug use, 16 had multidrug-resistant tuberculosis, 25 were underweight, and 51 had no HIV test performed. Crude outcome distributions by covariate are presented in Table S4, and observed distributions for selected exposures are shown in Figure S2.

Patients who died had a baseline distribution different from that observed for LTFU. In this group, 17 of 30 were 38 years or older, 10 were underweight, five had multidrug-resistant tuberculosis, and six were HIV positive. In the not evaluated/transferred category, 18 of 60 patients had multidrug-resistant tuberculosis and 17 had no HIV test performed.

### 3.3. Primary Multinomial Model

In the adjusted multinomial model, associations varied according to the specific outcome category. For LTFU versus cure, the largest adjusted odds ratios (aORs) were observed for history of drug use, multidrug-resistant tuberculosis, HIV test not performed, underweight, weekly alcohol use, and incomplete secondary education. The estimates were 4.15 for history of drug use, 4.58 for multidrug-resistant tuberculosis, 2.51 for HIV test not performed, 2.19 for underweight, 2.12 for weekly alcohol use, and 1.77 for incomplete secondary education. Ages 38 years or older showed a lower aOR for LTFU versus cure, with an estimate of 0.50. These results are presented graphically in Figure 2, and the complete numeric values are reported in Table S5.

For death versus cure, the association pattern differed. Death was infrequent (*n* = 30), HIV-positive status was uncommon (*n* = 22), and six deaths occurred among HIV-positive participants. HIV-positive status showed the largest adjusted association with death in the ridge-penalized model (aOR 32.12; 95% CI 8.95–115.33), although the estimate was imprecise. Elevated estimates were also observed for MDR-TB (aOR 5.04; 95% CI 1.62–15.69), underweight (aOR 3.77; 95% CI 1.57–9.03), HIV testing not performed (aOR 3.19; 95% CI 1.37–7.44), and incomplete secondary education (aOR 2.46; 95% CI 1.06–5.70). History of drug use was not associated with higher odds of death (aOR 0.46; 95% CI 0.12–1.77).

For not evaluated/transferred versus cured, the largest association was observed for MDR-TB (aOR 11.40; 95% CI 5.86–22.19). HIV testing not performed was also associated with this category (aOR 2.01; 95% CI 1.08–3.74). Component-specific analyses indicated that the MDR-TB estimate for the combined category was largely driven by participants still in treatment at database closure (aOR 49.92; 95% CI 18.06–138.01), whereas the estimate for transfer was smaller (aOR 3.17; 95% CI 1.04–9.66). This pattern is consistent with treatment duration and database closure effects, without establishing that these mechanisms caused the association.

Approximate Wald tests showed differences across outcome categories for history of drug use and HIV-positive status. Heterogeneity was also observed for the age category of 38 years or older, whereas the underweight test was at the conventional threshold. These tests support non-interchangeability of several outcome-specific estimates but should be interpreted together with effect magnitudes and confidence intervals rather than as a binary significance screen.

### 3.4. Composite Outcomes

In binary models of composite outcomes, associations depended on the definition used. For absence of a documented cure, excluding ongoing treatment versus cured, the highest adjusted odds corresponded to MDR-TB, HIV-positive status, history of drug use, HIV testing not performed, underweight, weekly alcohol use, and incomplete secondary education. The estimates were 4.40 for MDR-TB, 3.93 for HIV-positive status, 2.76 for history of drug use, 2.61 for HIV testing not performed, 2.04 for underweight, 1.78 for weekly alcohol use, and 1.60 for incomplete secondary education.

For the strict unfavorable outcome, defined as LTFU or death versus cure, adjusted odds were 5.06 for HIV-positive status, 4.64 for MDR-TB, 3.19 for history of drug use, 2.64 for HIV testing not performed, 2.57 for underweight, 1.89 for incomplete secondary education, and 1.85 for weekly alcohol use. These composite estimates represent aggregate endpoints rather than a single clinical mechanism. A covariate strongly associated with one component may appear attenuated or amplified when combined with outcomes governed by different processes. Although aggregation increased the total number of events, it did not eliminate sparse information within specific outcome–exposure cells; in particular, the HIV association with the strict unfavorable endpoint remained partly influenced by the same six HIV-positive deaths. The results for both composite outcomes are shown in Table 2.

### 3.5. Sensitivity Analyses

Unadjusted outcome-specific associations were consistent with the overall pattern observed in the adjusted model, although they were more influenced by uncontrolled baseline differences, as shown in Table S6. In replication contrasts of the binary LTFU analysis, history of drug use, multidrug-resistant tuberculosis, HIV test not performed, underweight, weekly alcohol use, and incomplete secondary education maintained higher adjusted odds versus the reference groups. In contrast to LTFU versus all other outcomes, adjusted odds were 4.13 for history of drug use, 2.97 for multidrug-resistant tuberculosis, 2.31 for HIV test not performed, 2.10 for weekly alcohol use, 2.09 for underweight, and 1.70 for incomplete secondary education. These results are presented in Table S7.

Sensitivity analyses of missing data did not materially change the direction of the main estimates. Estimates for history of drug use, MDR-TB, HIV testing not performed, underweight, and weekly alcohol use remained in the same direction for LTFU versus cure when the analytic denominator was expanded and when the single stochastic hot-deck imputation sensitivity analysis was applied (Table S8). The hot-deck analysis was interpreted as a directional check and did not incorporate between-imputation uncertainty.

Sensitivity analysis of the not evaluated/transferred category showed that MDR-TB maintained a high association with outcomes related to an absence of documented cure. When the components were separated, the aOR was 49.92 (95% CI 18.06–138.01) for still in treatment and 3.17 (95% CI 1.04–9.66) for transfer, confirming that the combined estimate was largely driven by ongoing treatment at database closure (Table S9). The extended model with additional baseline covariates preserved the overall pattern of associations, although several estimates were less precise (Table S10).

The mean bias-reduced multinomial model produced estimates with the same overall direction and ordering as the ridge-penalized primary model (Table S11). For HIV-positive status and death versus cure, the bias-reduced aOR was 35.56 (95% CI 9.80–129.08) compared with 32.12 (95% CI 8.95–115.33) in the primary model. Thus, the large association persisted but remained imprecisely estimated and sensitive in magnitude to model specification. Complete adjusted estimates are provided in Table S5; missing-data and nonterminal-outcome sensitivities are shown in Table S8 and Table S9, respectively; and standardized absolute probabilities are presented in Figure S3.

Standardized marginal probabilities provided an absolute-scale description. For history of drug use, the standardized probability of LTFU was 22.0% in the exposed scenario and 7.0% in the reference scenario. For multidrug-resistant tuberculosis, the standardized probability of a cure was 51.2% in the exposed scenario and 84.5% in the reference scenario, whereas the probability of not evaluated/transferred was 23.1% and 3.7%, respectively. For HIV-positive status, the standardized probability of death was 24.2% in the exposed scenario and 2.0% in the reference scenario. These estimates are presented in Figure S3. Figure S4 summarizes the demographic, social, behavioral, and clinical domains used to organize the covariates.

## 4. Discussion

### 4.1. Main Findings

In this secondary analysis, the endpoint definition modified the profile of associations observed with antituberculosis treatment outcomes. History of drug use, weekly alcohol use, underweight, MDR-TB, HIV testing not performed, and incomplete secondary education were associated with LTFU versus cure, but the pattern was not equivalent for death or not evaluated/transferred. HIV-positive status showed the largest—although imprecisely estimated—association with death, whereas the association between MDR-TB and the combined not evaluated/transferred category was largely driven by participants still in treatment at database closure. Composite odds ratios reflect mixtures of component-specific processes whose contribution depended on the frequency and covariate structure of each component; they should not be interpreted as estimates of a single clinical mechanism. These findings show that binary collapsing can materially change epidemiologic inference in this cohort [8,9,10].

### 4.2. Comparison with the Literature

The pattern observed for LTFU is consistent with previous studies linking treatment interruption to behavioral and social factors. In the original Lima study, drug use, weekly alcohol use, underweight, multidrug-resistant tuberculosis, HIV test not performed, and incomplete secondary education were associated with default in a binary model [8]. A systematic review from developing countries identified socioeconomic, behavioral, and health service-related factors as common determinants of non-adherence and loss to follow-up [17]. In Brazil, Garrido and colleagues reported associations between default and a low educational level, alcoholism, TB/HIV coinfection, and comorbidities, whereas in South Africa, Kigozi and colleagues described a higher probability of default in younger groups and in patients with socially adverse conditions [18,19]. Similarly, a systematic review with a meta-analysis found that not consuming alcohol and being HIV-negative were associated with a higher probability of treatment success [20]. These antecedents are consistent with the behavioral and social components of LTFU observed in the present analysis. However, reported associations for age, education, alcohol use, and HIV have not been uniform across settings [17,18,19,20,21,22,23]. Such differences may reflect genuine contextual variations, but they may also arise from non-equivalent endpoint definitions, treatment regimens, follow-up windows, and covariate adjustment sets, which limit a direct comparison across studies.

The association of MDR-TB with LTFU, not evaluated/transferred, and absence of documented cure is consistent with the historical treatment context of the cohort. MDR/RR-TB regimens used during 2010–2011 were generally longer and more burdensome than contemporary all-oral regimens, which may have increased opportunities for interruption, transfer, prolonged treatment, or an unresolved outcome at database closure. Component-specific analyses in the present study support this interpretation because the association with still in treatment was substantially larger than the association with transfer. Contemporary WHO guidance now prioritizes shorter all-oral regimens for eligible patients [6]; therefore, these historical estimates should not be transported directly to current practice. Prior studies, nevertheless, document heterogeneous adverse outcomes during drug-resistant tuberculosis treatment, including LTFU and death [24,25,26].

The specific association between HIV-positive status and death, rather than LTFU, is consistent with Peruvian and global evidence on mortality during antituberculosis treatment. In a prospective cohort from Lima, Velasquez and colleagues reported that patients with HIV had higher mortality during tuberculosis treatment and a lower proportion of being cured, even in a national context of low HIV prevalence [21]. Among patients with HIV/MDR-TB coinfection treated in Lima, Palacios and colleagues reported high mortality during treatment, with a lower risk of death among those receiving highly active antiretroviral therapy and a higher risk associated with a low baseline weight [22]. A recent systematic review of tuberculosis mortality in South Africa identified HIV infection, drug-resistant tuberculosis, and a lower body weight at diagnosis as factors associated with death during treatment [23]. Together, these studies support that an HIV-positive status, being underweight, and drug resistance are related to severe clinical outcomes, whereas HIV tests not performed may represent a programmatic marker of access, recording, or continuity of care. In the present cohort, however, only six deaths occurred among HIV-positive participants; the direction and large magnitude of the association were stable in bias-reduced analyses, but its exact size remains uncertain.

The methodological contribution of this study lies in the international debate on the definition of antituberculosis treatment outcomes. The WHO has standardized categories such as cured, treatment completed, treatment failed, died, loss to follow-up, and not evaluated to enable comparability across programs [7]. Before the recent updates, Laserson and colleagues had already emphasized the need to speak the same language in multidrug-resistant tuberculosis outcomes [27]. Subsequently, the 2021 update of WHO outcome definitions sought to make them more uniformly applicable to regimens of different durations and resistance profiles [12]. In addition, recent methodological reviews have noted that the validity of composite outcomes depends on the clinical coherence of grouped categories and the ability to compare results across programs and studies [28]. Our results provide empirical evidence from a Latin American cohort: endpoint selection is not a semantic detail but a decision that changes epidemiologic inference. The present analyses further show that composite estimates are frequency-weighted mixtures of component-specific associations and can obscure clinically or programmatically important differences.

### 4.3. Implications for Public and International Health

From a programmatic perspective, these findings suggest that monitoring tuberculosis outcomes should not be limited to aggregate indicators of treatment success or non-success. In high-burden urban settings such as Lima and Callao, a substantial proportion of tuberculosis, MDR-TB, and XDR-TB cases are territorially concentrated, and outcome categories may reflect different problems: treatment interruptions, clinical lethality, prolonged therapy, transfers, or an absence of a final evaluation [4,5]. For national and international surveillance, reporting only composite outcomes may be insufficient to guide interventions. A reduction in LTFU does not necessarily imply lower mortality, and a higher proportion of not-evaluated patients may reflect failures in traceability rather than individual therapeutic failure. For example, a composite indicator driven predominantly by LTFU may direct attention toward retention strategies, patient navigation, and social support, whereas the same aggregate indicator driven predominantly by mortality may instead prioritize clinical monitoring, HIV care, and management of disease severity. Similarly, an excess of transferred or unevaluated records may signal a need to strengthen interfacility information linkages and outcome traceability rather than adherence counseling.

These examples are interpretive rather than causal intervention effects. Associations of drug use, alcohol use, and incomplete education with LTFU identify domains that may warrant evaluation, but they do not establish that targeting those factors will improve treatment completion. Evidence from adherence interventions suggests that integrated packages of education, counseling, incentives, psychological support, reminders, and tracing may improve outcomes, whereas direct observation alone is insufficient [29,30]. Prospective or experimental evaluation is required before assigning a specific intervention to a subgroup on the basis of the present associations.

At the international level, outcome heterogeneity is relevant to the End TB Strategy, which emphasizes integrated patient-centered care, supportive policies, research, and innovation [31]. The WHO and ILO have noted that social protection is part of the response to tuberculosis and that programs should address the direct costs, income loss, and social barriers affecting continuity of care [32]. The present analysis translates that agenda into a measurable problem: outcome categories do not merely count events; they also reveal which type of programmatic or clinical failure predominates in each subgroup. This approach may be transferable to other low- and middle-income countries, particularly in urban areas with concentrations of poverty, population mobility, drug-resistant tuberculosis, and HIV coinfection.

### 4.4. Limitations

This study has several limitations. First, it is a secondary analysis of a historical cohort enrolled in 2010–2011. Contemporary diagnostic pathways, HIV care, and MDR/RR-TB regimens differ substantially [6]; therefore, these findings are best interpreted as a methodological demonstration of endpoint sensitivity rather than as current risk estimates for Lima. Second, the available baseline associations do not support causal inference because longitudinal exposure measurements, detailed disease severity indicators, and comparable follow-up times across all outcomes were unavailable. Third, several covariates were coarsely categorized, preventing more flexible modeling of age, body mass index, alcohol use, and drug use. Fourth, death was infrequent (*n* = 30), HIV-positive status was uncommon (*n* = 22), and only six deaths occurred among HIV-positive participants. Penalization reduced numerical instability but did not add information to sparse outcome–exposure cells; consequently, some estimates, particularly the HIV–death association, remain imprecise and model-sensitive. The results were similar in a mean bias-reduced multinomial sensitivity analysis. Fifth, transferred and still-in-treatment records both lacked a documented final outcome at the source facility but represent conceptually different states; the latter is nonterminal and may partly reflect treatment duration and database closure. We therefore evaluated definitions that excluded ongoing treatment and separated both components. Finally, complete-case analysis may be biased under nonrandom missingness. The single stochastic hot-deck analysis provided a directional sensitivity check but did not account for between-imputation uncertainties.

## 5. Conclusions

In this historical cohort of adults with smear-positive pulmonary tuberculosis, baseline associations differed across mutually exclusive treatment–outcome categories. Behavioral and social vulnerability factors were more prominent for LTFU, whereas HIV-positive status showed the largest—although imprecisely estimated—association with death, and MDR-TB was associated with several forms of absence of a documented cure or final outcome. Composite endpoints altered the magnitude and interpretation of these associations and should not be regarded as clinically homogeneous.

These findings support reporting disaggregated treatment–outcome categories alongside any composite indicator when data permit. They should not be interpreted as contemporary risk estimates for Lima or as evidence that a specific intervention will improve treatment outcomes. Future studies should evaluate the same endpoint architecture in contemporary cohorts with complete follow-up times, serial bacteriological data, antiretroviral therapy and adverse-event information, health facility characteristics, and measures of social support and interfacility outcome traceability.

## Figures and Tables

**Figure 1:**
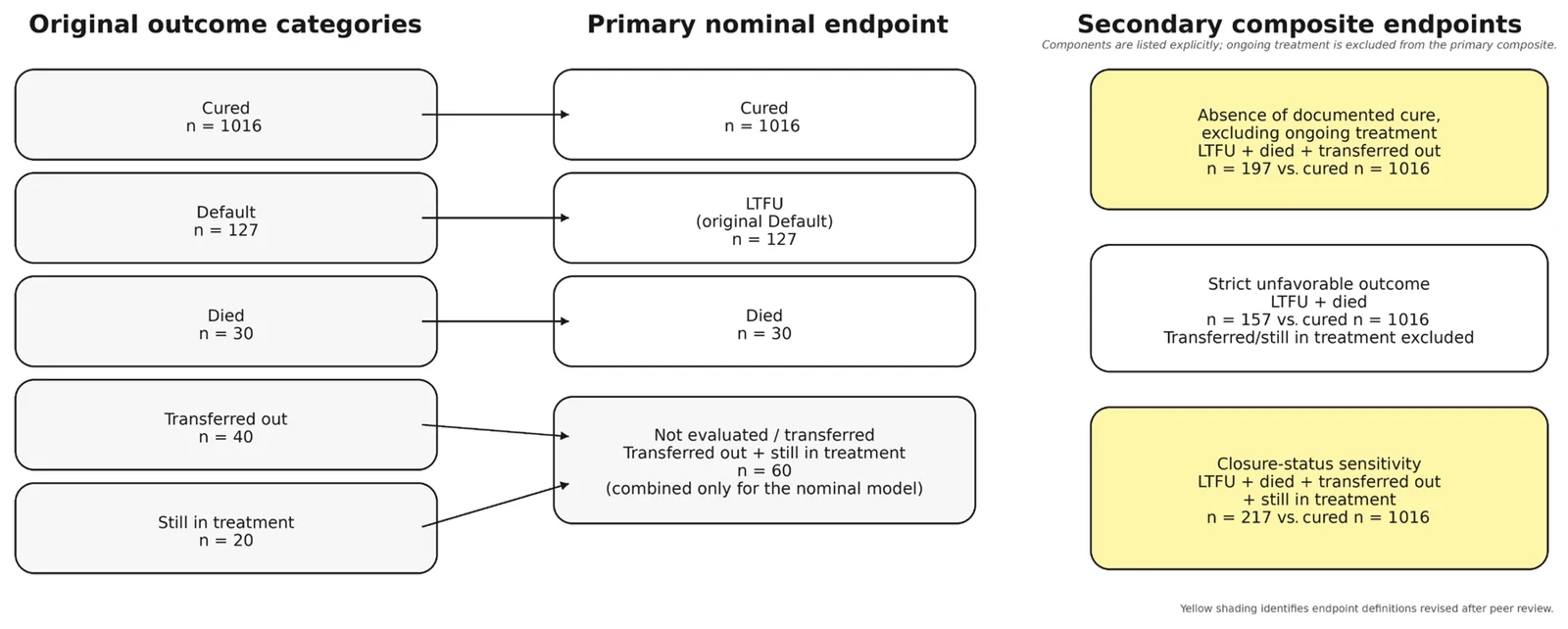
Revised outcome architecture for the analysis of treatment–outcome heterogeneity. LTFU = loss to follow-up. The source dataset labels this category as “Default”. The primary composite excludes participants still in treatment, whereas the broader closure-status definition is retained only as a sensitivity analysis. Yellow shading identifies endpoint definitions revised in response to peer review.

**Figure 2:**
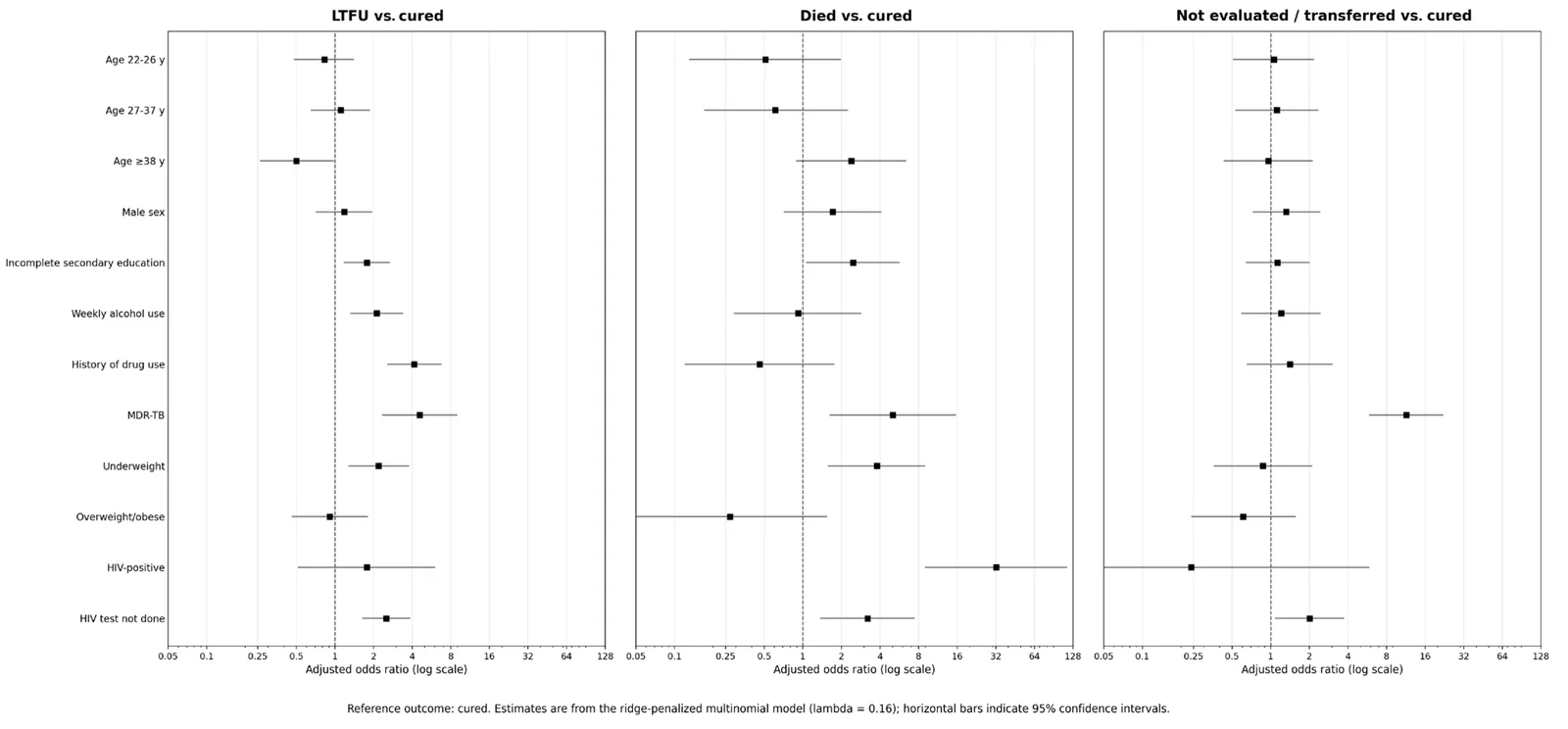
Adjusted associations according to the primary multinomial outcome. Adjusted odds ratios (aORs) were obtained using a ridge-penalized multinomial model with lambda = 0.16; “Cured” is the reference outcome. Horizontal bars indicate 95% confidence intervals.

**Table 1:** Baseline characteristics according to the primary treatment–outcome category.

Characteristic	Total *n* = 1233	Cured *n* = 1016	LTFU *n* = 127	Died *n* = 30	Not Evaluated/Transferred *n* = 60
**Age group**	**Age group**	**Age group**	**Age group**	**Age group**	**Age group**
21 or younger	321 (26.0)	263 (25.9)	37 (29.1)	6 (20.0)	15 (25.0)
22 to 26	326 (26.4)	270 (26.6)	36 (28.3)	3 (10.0)	17 (28.3)
27 to 37	291 (23.6)	233 (22.9)	39 (30.7)	4 (13.3)	15 (25.0)
38 or older	295 (23.9)	250 (24.6)	15 (11.8)	17 (56.7)	13 (21.7)
**Sex**	**Sex**	**Sex**	**Sex**	**Sex**	**Sex**
Female	490 (39.7)	433 (42.6)	29 (22.8)	9 (30.0)	19 (31.7)
Male	743 (60.3)	583 (57.4)	98 (77.2)	21 (70.0)	41 (68.3)
**Marital status**	**Marital status**	**Marital status**	**Marital status**	**Marital status**	**Marital status**
Single	637 (51.7)	525 (51.7)	70 (55.1)	11 (36.7)	31 (51.7)
Married or cohabiting	469 (38.0)	391 (38.5)	45 (35.4)	9 (30.0)	24 (40.0)
Divorced or separated	91 (7.4)	72 (7.1)	10 (7.9)	5 (16.7)	4 (6.7)
Widowed	36 (2.9)	28 (2.8)	2 (1.6)	5 (16.7)	1 (1.7)
**Poverty status**	**Poverty status**	**Poverty status**	**Poverty status**	**Poverty status**	**Poverty status**
No poverty	1024 (83.0)	852 (83.9)	97 (76.4)	24 (80.0)	51 (85.0)
Poverty/extreme poverty	209 (17.0)	164 (16.1)	30 (23.6)	6 (20.0)	9 (15.0)
**History of imprisonment**	**History of imprisonment**	**History of imprisonment**	**History of imprisonment**	**History of imprisonment**	**History of imprisonment**
No	1178 (95.5)	984 (96.9)	109 (85.8)	29 (96.7)	56 (93.3)
Yes	55 (4.5)	32 (3.1)	18 (14.2)	1 (3.3)	4 (6.7)
**Completed secondary education**	**Completed secondary education**	**Completed secondary education**	**Completed secondary education**	**Completed secondary education**	**Completed secondary education**
Yes	719 (58.3)	622 (61.2)	52 (40.9)	10 (33.3)	35 (58.3)
No	514 (41.7)	394 (38.8)	75 (59.1)	20 (66.7)	25 (41.7)
**Tobacco use**	**Tobacco use**	**Tobacco use**	**Tobacco use**	**Tobacco use**	**Tobacco use**
Never smoked	742 (60.2)	638 (62.8)	50 (39.4)	18 (60.0)	36 (60.0)
Former smoker	440 (35.7)	337 (33.2)	69 (54.3)	12 (40.0)	22 (36.7)
Current smoker	51 (4.1)	41 (4.0)	8 (6.3)	0 (0.0)	2 (3.3)
**Alcohol use at least once per week**	**Alcohol use at least once per week**	**Alcohol use at least once per week**	**Alcohol use at least once per week**	**Alcohol use at least once per week**	**Alcohol use at least once per week**
No	1000 (81.1)	855 (84.2)	72 (56.7)	26 (86.7)	47 (78.3)
Yes	233 (18.9)	161 (15.8)	55 (43.3)	4 (13.3)	13 (21.7)
**History of drug use**	**History of drug use**	**History of drug use**	**History of drug use**	**History of drug use**	**History of drug use**
No	1043 (84.6)	903 (88.9)	64 (50.4)	27 (90.0)	49 (81.7)
Yes	190 (15.4)	113 (11.1)	63 (49.6)	3 (10.0)	11 (18.3)
**History of rehabilitation**	**History of rehabilitation**	**History of rehabilitation**	**History of rehabilitation**	**History of rehabilitation**	**History of rehabilitation**
No	1156 (93.8)	970 (95.5)	102 (80.3)	29 (96.7)	55 (91.7)
Yes	77 (6.2)	46 (4.5)	25 (19.7)	1 (3.3)	5 (8.3)
**MDR-TB**	**MDR-TB**	**MDR-TB**	**MDR-TB**	**MDR-TB**	**MDR-TB**
No	1152 (93.4)	974 (95.9)	111 (87.4)	25 (83.3)	42 (70.0)
Yes	81 (6.6)	42 (4.1)	16 (12.6)	5 (16.7)	18 (30.0)
**Body mass index**	**Body mass index**	**Body mass index**	**Body mass index**	**Body mass index**	**Body mass index**
Normal	916 (74.3)	757 (74.5)	91 (71.7)	19 (63.3)	49 (81.7)
Underweight	151 (12.2)	110 (10.8)	25 (19.7)	10 (33.3)	6 (10.0)
Overweight/obesity	166 (13.5)	149 (14.7)	11 (8.7)	1 (3.3)	5 (8.3)
**History of chronic disease**	**History of chronic disease**	**History of chronic disease**	**History of chronic disease**	**History of chronic disease**	**History of chronic disease**
No	1198 (97.2)	986 (97.0)	125 (98.4)	28 (93.3)	59 (98.3)
Yes	35 (2.8)	30 (3.0)	2 (1.6)	2 (6.7)	1 (1.7)
**HIV status**	**HIV status**	**HIV status**	**HIV status**	**HIV status**	**HIV status**
Negative	916 (74.3)	788 (77.6)	72 (56.7)	13 (43.3)	43 (71.7)
Positive	22 (1.8)	12 (1.2)	4 (3.1)	6 (20.0)	0 (0.0)
Test not performed	295 (23.9)	216 (21.3)	51 (40.2)	11 (36.7)	17 (28.3)
**History of diabetes mellitus**	**History of diabetes mellitus**	**History of diabetes mellitus**	**History of diabetes mellitus**	**History of diabetes mellitus**	**History of diabetes mellitus**
No	1179 (95.6)	969 (95.4)	127 (100.0)	28 (93.3)	55 (91.7)
Yes	54 (4.4)	47 (4.6)	0 (0.0)	2 (6.7)	5 (8.3)

Values are expressed as *n* (%); *p*-values are not reported because the table is descriptive. The distribution across rows within each outcome category uses the denomination LTFU for the category programmatically recorded as default. LTFU = loss to follow-up.

**Table 2:** Composite binary outcome models.

Predictor	Absence of Documented Cure, Excluding Ongoing Treatment, vs. Cured, aOR (95% CI)	Absence of Documented Cure, Excluding Ongoing Treatment, vs. Cured, *p*	Strict Unfavorable Outcome (LTFU or Death) vs. Cured, aOR (95% CI)	Strict Unfavorable Outcome vs. Cured, *p*
Age 22–26 vs. ≤21	0.83 (0.52–1.32)	0.433	0.76 (0.46–1.27)	0.301
Age 27–37 vs. ≤21	1.10 (0.69–1.74)	0.686	1.05 (0.63–1.74)	0.853
Age ≥38 vs. ≤21	0.95 (0.59–1.55)	0.835	0.85 (0.49–1.47)	0.564
Weekly alcohol use vs. no	1.78 (1.18–2.68)	0.006	1.85 (1.18–2.90)	0.008
HIV positive vs. negative	3.93 (1.54–10.06)	0.004	5.06 (1.93–13.26)	<0.001
HIV test not performed vs. negative	2.61 (1.82–3.74)	<0.001	2.64 (1.77–3.95)	<0.001
History of drug use vs. no	2.76 (1.81–4.22)	<0.001	3.19 (2.01–5.06)	<0.001
Incomplete secondary education vs. complete	1.60 (1.13–2.26)	0.008	1.89 (1.29–2.78)	0.001
MDR-TB vs. no	4.40 (2.45–7.88)	<0.001	4.64 (2.45–8.78)	<0.001
Male vs. female	1.36 (0.92–2.03)	0.128	1.32 (0.84–2.06)	0.232
Overweight/obesity vs. normal BMI	0.69 (0.39–1.22)	0.202	0.70 (0.37–1.35)	0.290
Underweight vs. normal BMI	2.04 (1.29–3.22)	0.002	2.57 (1.58–4.18)	<0.001

Values correspond to adjusted odds ratios with 95% confidence intervals derived from ridge-penalized logistic regression with lambda = 0.16. The absence-of-documented-cure endpoint includes LTFU, death, and transferred out and excludes participants still in treatment at database closure. The strict unfavorable endpoint includes only LTFU and death versus cure. Composite estimates should not be interpreted as reflecting a single clinical mechanism.

## Data Availability

The microdata used in this study are publicly available at: https://datadryad.org/dataset/doi:10.5061/dryad.fp94d (accessed on 11 May 2026).

## Supplementary Materials

The following supporting information can be downloaded at: https://www.mdpi.com/article/10.3390/idr18050108/s1, Table S1: STROBE checklist for cohort studies; Table S2: Variable dictionary and analytic recoding; Table S3: Missing baseline data among patients with recorded outcome; Table S4: Crude outcome distribution by covariate level; Table S5: Primary ridge-penalized multinomial model—complete adjusted estimates; Table S6: Unadjusted outcome-specific associations; Table S7: Replication of the original contrasts for LTFU; Table S8: Missing-data sensitivity analysis, including a single stochastic hot-deck imputation; Table S9: Sensitivity analysis for transferred/still-in-treatment definitions; Table S10: Extended model, including all baseline covariates; Table S11: Sparse-data sensitivity analysis comparing ridge-penalized and mean bias-reduced multinomial estimates; Figure S1: Participant flow diagram; Figure S2: Observed outcome distribution according to key exposures; Figure S3: Standardized marginal probabilities in selected exposure scenarios; Figure S4: Conceptual framework by covariate domains.
