# Supplementary material for: How Endpoint Definitions Shape Epidemiologic Inference on Tuberculosis Treatment Outcomes: A Secondary Analysis from Lima, Peru
Source: Infect Dis Rep. 2026 Sep 26;18(5):108. doi: 10.3390/idr18050108 (PMC13704616; doi:10.3390/idr18050108)

Supplementary Figure S1. Participant flow diagram.

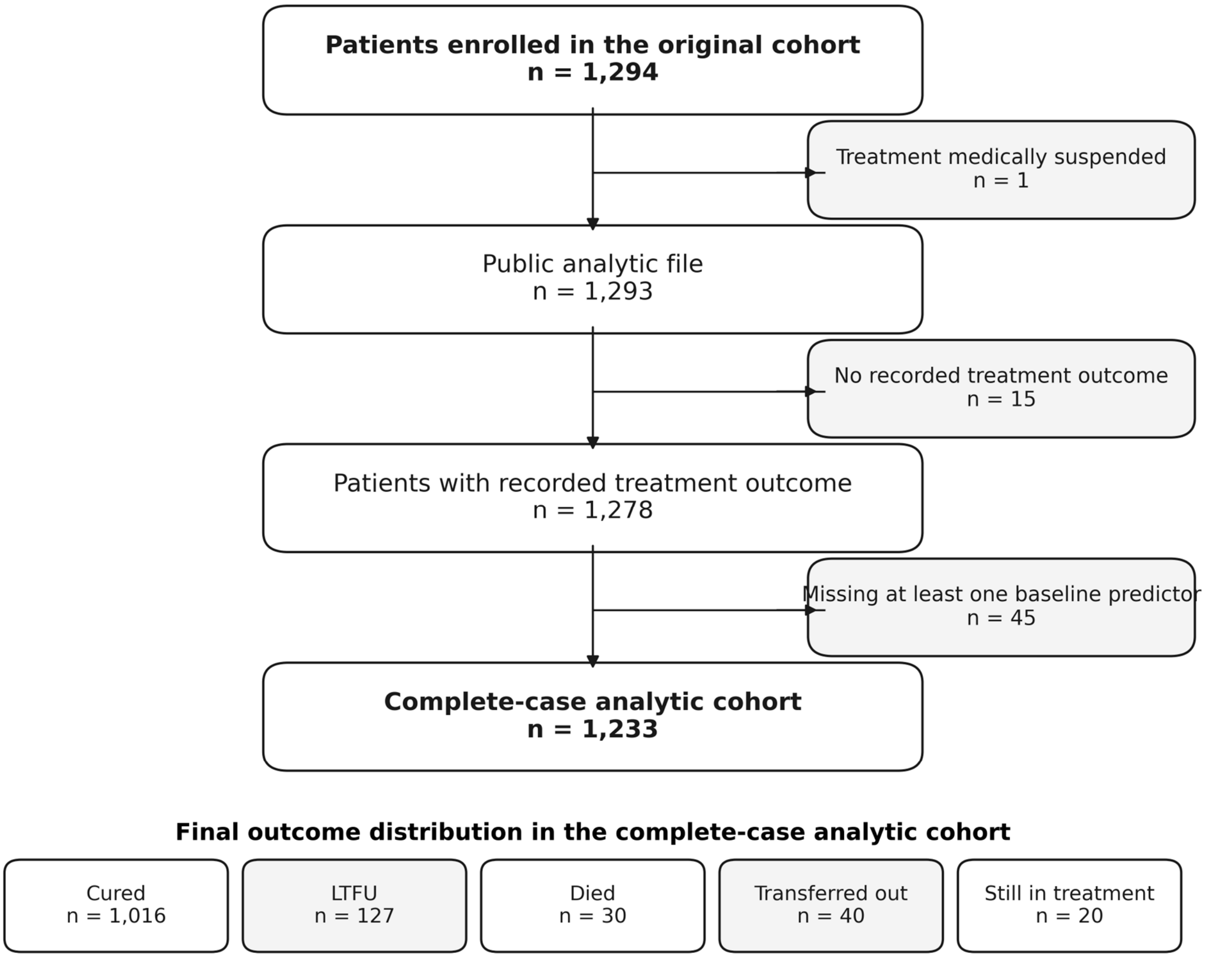

Supplement: Supplementary file 1 [file idr-18-00108-s001.zip › Figure_S1_Flow.pdf]
