# Supplementary material for: How Endpoint Definitions Shape Epidemiologic Inference on Tuberculosis Treatment Outcomes: A Secondary Analysis from Lima, Peru
Source: Infect Dis Rep. 2026 Sep 26;18(5):108. doi: 10.3390/idr18050108 (PMC13704616; doi:10.3390/idr18050108)

Supplementary Figure S2. Observed outcome distribution according to key exposures. Descriptive visualization (stacked bar charts) of percentages by outcome category for the predictors with the greatest statistical contribution: MDR-TB, history of drug use, weekly alcohol use, and HIV status.

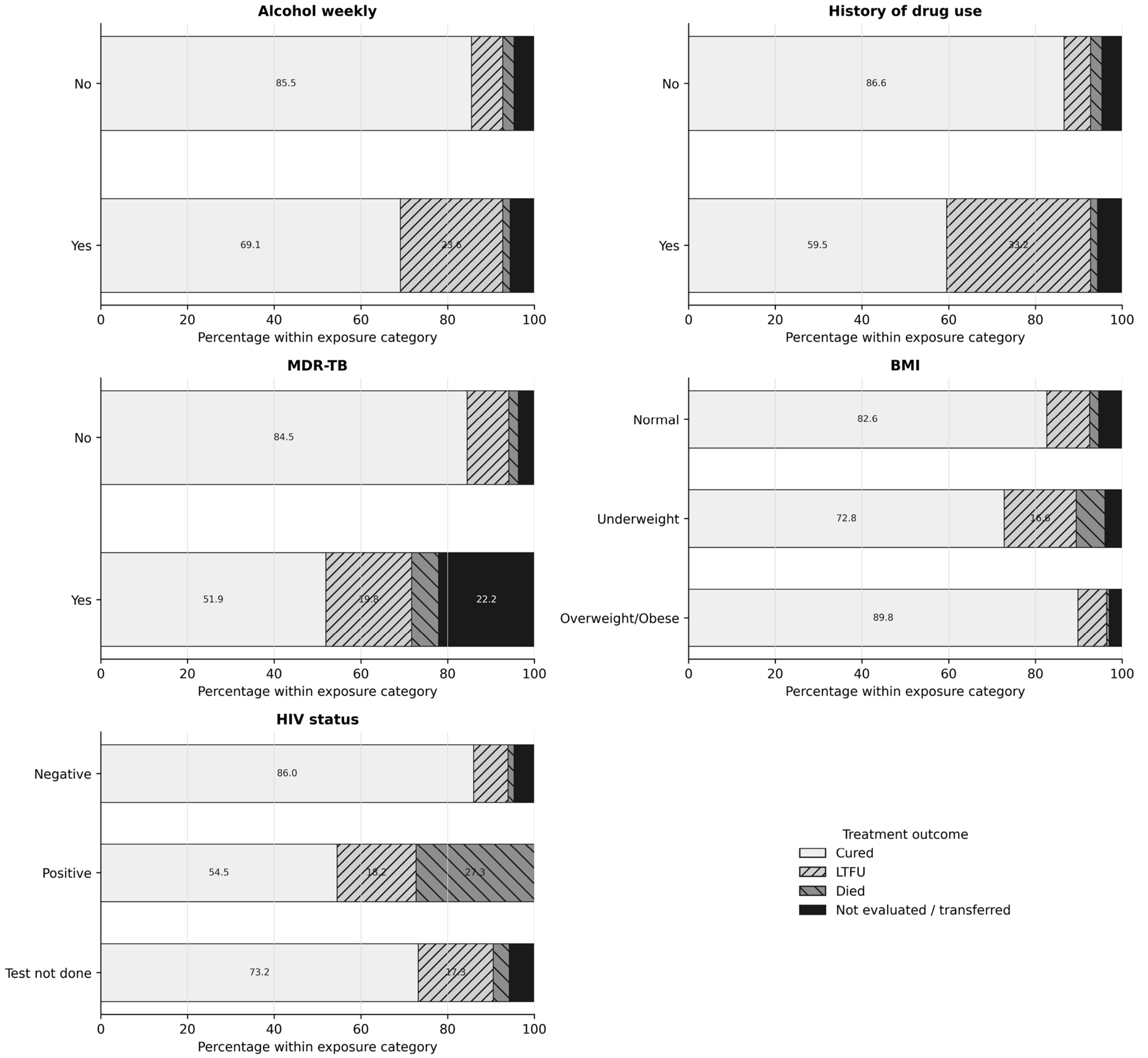

Supplement: Supplementary file 1 [file idr-18-00108-s001.zip › Figure_S2_OutcomeDistribution.pdf]
