# Supplementary material for: How Endpoint Definitions Shape Epidemiologic Inference on Tuberculosis Treatment Outcomes: A Secondary Analysis from Lima, Peru
Source: Infect Dis Rep. 2026 Sep 26;18(5):108. doi: 10.3390/idr18050108 (PMC13704616; doi:10.3390/idr18050108)

Supplementary Figure S3. Standardized marginal probabilities in selected exposure scenarios. Marginal probabilities predicted by the primary multinomial model for counterfactual exposure scenarios, standardized to the empirical distribution of the remaining covariates.

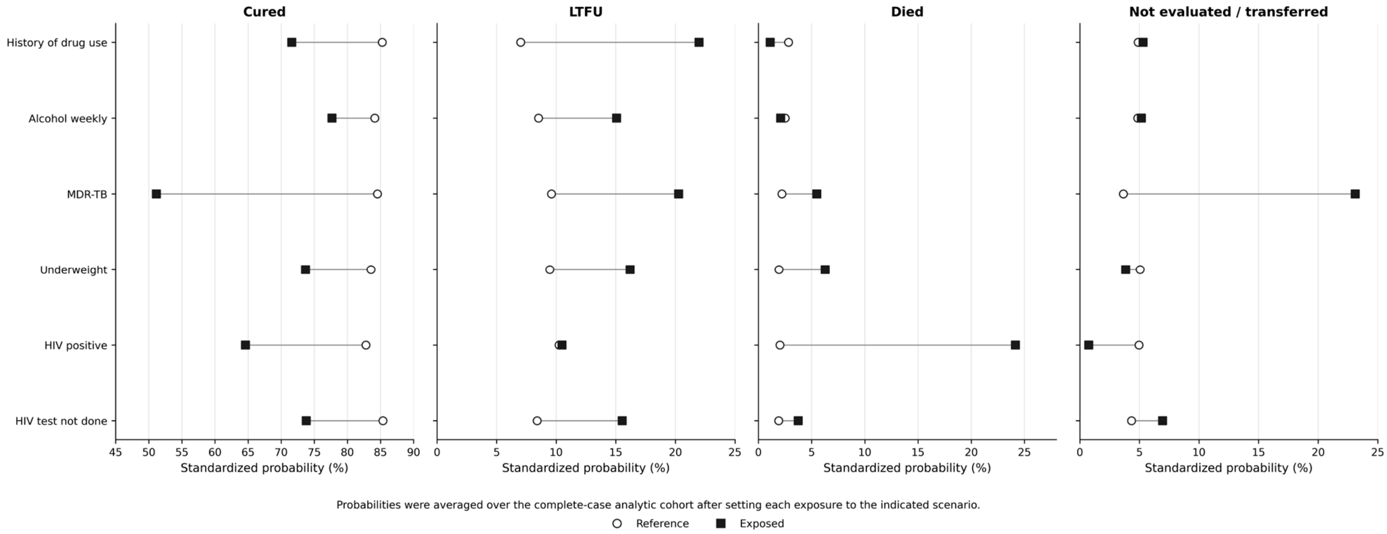

Supplement: Supplementary file 1 [file idr-18-00108-s001.zip › Figure_S3_StandardizedProb.pdf]
