# Supplementary material for: How Endpoint Definitions Shape Epidemiologic Inference on Tuberculosis Treatment Outcomes: A Secondary Analysis from Lima, Peru
Source: Infect Dis Rep. 2026 Sep 26;18(5):108. doi: 10.3390/idr18050108 (PMC13704616; doi:10.3390/idr18050108)

Supplementary Figure S4. Conceptual framework by covariate domains. Conceptual diagram organizing covariates into four domains: demographic (age, sex), social (education, poverty, imprisonment, marital status), behavioral (alcohol, tobacco, drugs, rehabilitation), and clinical (HIV, MDR-TB, BMI, chronic disease, diabetes).

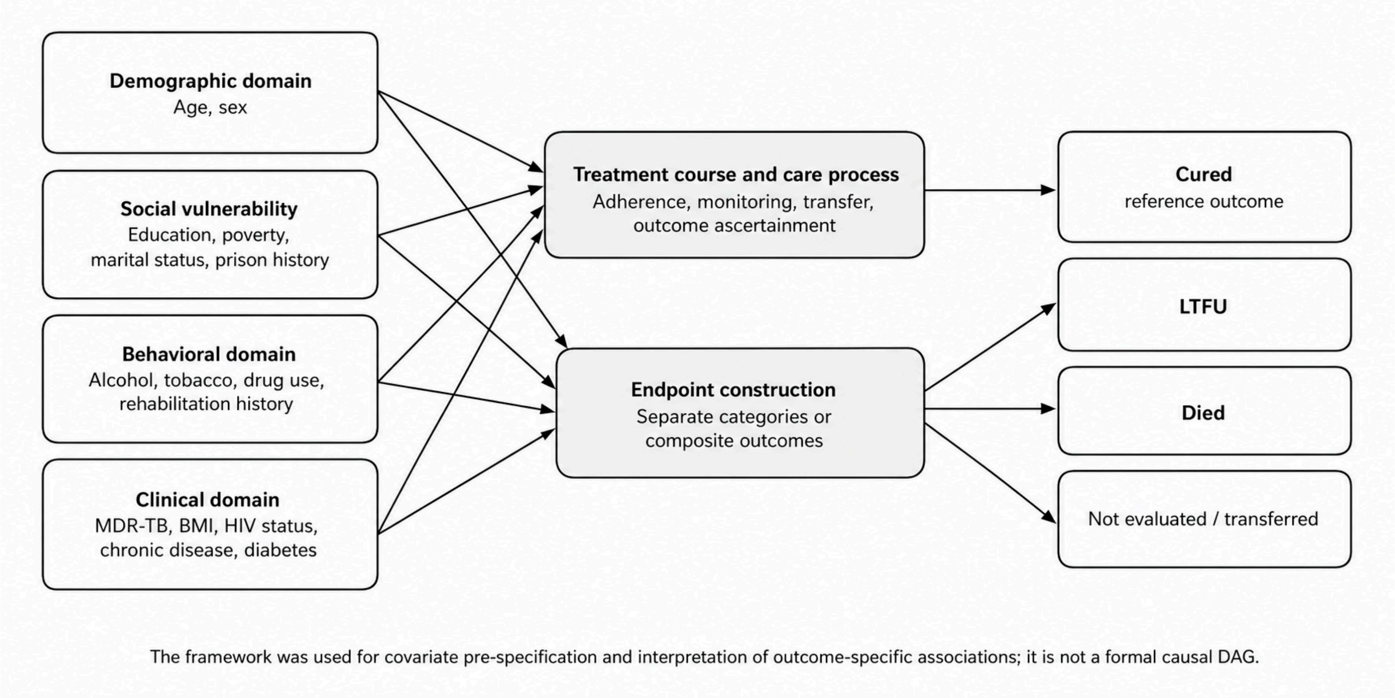

Supplement: Supplementary file 1 [file idr-18-00108-s001.zip › Figure_S4_ConceptualFramework.pdf]
