# Supplementary material for: How Endpoint Definitions Shape Epidemiologic Inference on Tuberculosis Treatment Outcomes: A Secondary Analysis from Lima, Peru
Source: Infect Dis Rep. 2026 Sep 26;18(5):108. doi: 10.3390/idr18050108 (PMC13704616; doi:10.3390/idr18050108)
Supplement: Supplementary file 1 [file idr-18-00108-s001.zip › Table_S10_Extended.pdf]

Supplementary Table S10. Extended model including all baseline covariates.

Outcome contrast	Predictor	aOR (95% CI)	p
LTFU vs Cured	Age 22-26 vs <=21	0.77 (0.45–1.33)	0.350
LTFU vs Cured	Age 27-37 vs <=21	0.99 (0.56–1.74)	0.969
LTFU vs Cured	Age >=38 vs <=21	0.49 (0.24–1.00)	0.051
LTFU vs Cured	Male vs female	1.18 (0.69–2.01)	0.552
LTFU vs Cured	Incomplete secondary education vs complete	1.61 (1.04–2.50)	0.033
LTFU vs Cured	Weekly alcohol use vs no	2.30 (1.39–3.81)	0.001
LTFU vs Cured	History of drug use vs no	4.20 (2.35–7.51)	<0.001
LTFU vs Cured	MDR-TB vs no	4.82 (2.42–9.59)	<0.001
LTFU vs Cured	Underweight vs normal BMI	2.19 (1.26–3.79)	0.005
LTFU vs Cured	Overweight/obesity vs normal BMI	0.92 (0.46–1.84)	0.809
LTFU vs Cured	HIV positive vs negative	1.86 (0.53–6.53)	0.330
LTFU vs Cured	HIV test not performed vs negative	2.58 (1.66–4.00)	<0.001
LTFU vs Cured	Married/cohabiting vs single	1.18 (0.73–1.89)	0.503
LTFU vs Cured	Divorced/separated vs single	1.05 (0.46–2.37)	0.916
LTFU vs Cured	Widowed vs single	1.36 (0.29–6.25)	0.696
LTFU vs Cured	Poverty/extreme poverty vs none	1.43 (0.86–2.38)	0.170
LTFU vs Cured	History of imprisonment vs no	1.69 (0.81–3.53)	0.161
LTFU vs Cured	Former smoker vs never smoked	0.89 (0.52–1.50)	0.656
LTFU vs Cured	Current smoker vs never smoked	0.50 (0.19–1.36)	0.176
LTFU vs Cured	History of rehabilitation vs no	0.95 (0.49–1.85)	0.892
LTFU vs Cured	Chronic disease vs no	0.86 (0.21–3.61)	0.842
LTFU vs Cured	Diabetes vs no	0.11 (0.01–1.80)	0.123
Died vs Cured	Age 22-26 vs <=21	0.56 (0.14–2.24)	0.415
Died vs Cured	Age 27-37 vs <=21	0.58 (0.15–2.31)	0.443
Died vs Cured	Age >=38 vs <=21	1.84 (0.57–5.96)	0.311
Died vs Cured	Male vs female	1.85 (0.71–4.82)	0.206
Died vs Cured	Incomplete secondary education vs complete	2.23 (0.91–5.48)	0.081
Died vs Cured	Weekly alcohol use vs no	0.96 (0.29–3.14)	0.945
Died vs Cured	History of drug use vs no	0.45 (0.10–2.00)	0.292
Died vs Cured	MDR-TB vs no	5.58 (1.75–17.84)	0.004
Died vs Cured	Underweight vs normal BMI	3.82 (1.56–9.32)	0.003
Died vs Cured	Overweight/obesity vs normal BMI	0.31 (0.05–1.86)	0.200
Died vs Cured	HIV positive vs negative	33.86 (8.97–127.89)	<0.001

Died vs Cured	HIV test not performed vs negative	3.57 (1.49–8.57)	0.004
Died vs Cured	Married/cohabiting vs single	0.83 (0.29–2.40)	0.730
Died vs Cured	Divorced/separated vs single	2.05 (0.54–7.69)	0.289
Died vs Cured	Widowed vs single	2.80 (0.65–12.19)	0.169
Died vs Cured	Poverty/extreme poverty vs none	1.03 (0.38–2.82)	0.949
Died vs Cured	History of imprisonment vs no	1.38 (0.15–12.63)	0.776
Died vs Cured	Former smoker vs never smoked	1.13 (0.45–2.81)	0.793
Died vs Cured	Current smoker vs never smoked	0.26 (0.01–7.03)	0.425
Died vs Cured	History of rehabilitation vs no	0.68 (0.08–5.82)	0.728
Died vs Cured	Chronic disease vs no	2.91 (0.59–14.40)	0.191
Died vs Cured	Diabetes vs no	1.37 (0.28–6.73)	0.700
Not evaluated/transferred vs Cured	Age 22-26 vs <=21	1.03 (0.49–2.16)	0.930
Not evaluated/transferred vs Cured	Age 27-37 vs <=21	1.03 (0.46–2.29)	0.941
Not evaluated/transferred vs Cured	Age >=38 vs <=21	0.81 (0.32–2.03)	0.652
Not evaluated/transferred vs Cured	Male vs female	1.34 (0.70–2.57)	0.371
Not evaluated/transferred vs Cured	Incomplete secondary education vs complete	1.10 (0.61–2.01)	0.749
Not evaluated/transferred vs Cured	Weekly alcohol use vs no	1.28 (0.61–2.68)	0.520
Not evaluated/transferred vs Cured	History of drug use vs no	1.30 (0.52–3.27)	0.580
Not evaluated/transferred vs Cured	MDR-TB vs no	11.12 (5.67–21.80)	<0.001
Not evaluated/transferred vs Cured	Underweight vs normal BMI	0.89 (0.36–2.17)	0.790
Not evaluated/transferred vs Cured	Overweight/obesity vs normal BMI	0.57 (0.22–1.49)	0.252
Not evaluated/transferred vs Cured	HIV positive vs negative	0.24 (0.01–5.99)	0.386
Not evaluated/transferred vs Cured	HIV test not performed vs negative	2.07 (1.11–3.86)	0.022
Not evaluated/transferred vs Cured	Married/cohabiting vs single	1.14 (0.60–2.18)	0.683
Not evaluated/transferred vs Cured	Divorced/separated vs single	1.00 (0.32–3.13)	0.996
Not evaluated/transferred vs Cured	Widowed vs single	0.85 (0.11–6.29)	0.871
Not evaluated/transferred vs Cured	Poverty/extreme poverty vs none	0.95 (0.44–2.05)	0.902

Not evaluated/transferred vs Cured	History of imprisonment vs no	1.62 (0.51–5.19)	0.414
Not evaluated/transferred vs Cured	Former smoker vs never smoked	0.88 (0.45–1.70)	0.695
Not evaluated/transferred vs Cured	Current smoker vs never smoked	0.61 (0.14–2.73)	0.523
Not evaluated/transferred vs Cured	History of rehabilitation vs no	1.21 (0.37–3.93)	0.750
Not evaluated/transferred vs Cured	Chronic disease vs no	0.48 (0.07–3.14)	0.445
Not evaluated/transferred vs Cured	Diabetes vs no	1.83 (0.60–5.56)	0.288

Saturated multinomial model including additional predictors (marital status, poverty, history of imprisonment, smoking, rehabilitation, chronic disease, and diabetes) added to the primary specification.
