# Supplementary material for: How Endpoint Definitions Shape Epidemiologic Inference on Tuberculosis Treatment Outcomes: A Secondary Analysis from Lima, Peru
Source: Infect Dis Rep. 2026 Sep 26;18(5):108. doi: 10.3390/idr18050108 (PMC13704616; doi:10.3390/idr18050108)
Supplement: Supplementary file 1 [file idr-18-00108-s001.zip › Table_S11_SparseDataSensitivity.pdf]

Supplementary Table S11. Sparse-data sensitivity analysis comparing ridge-penalized and mean bias-reduced multinomial estimates.

Outcome contrast	Predictor	Ridge lambda = 0.16, aOR (95% CI)	Mean bias-reduced aOR (95% CI)
LTFU vs cured	Age 22–26 vs ≤21	0.83 (0.48–1.41)	0.82 (0.48–1.40)
LTFU vs cured	Age 27–37 vs ≤21	1.11 (0.65–1.88)	1.10 (0.65–1.87)
LTFU vs cured	Age ≥38 vs ≤21	0.50 (0.26–0.98)	0.51 (0.26–0.99)
LTFU vs cured	Male vs female	1.18 (0.71–1.96)	1.17 (0.71–1.94)
LTFU vs cured	Incomplete secondary vs complete	1.77 (1.17–2.69)	1.76 (1.16–2.68)
LTFU vs cured	Weekly alcohol use vs no	2.12 (1.32–3.41)	2.12 (1.32–3.42)
LTFU vs cured	History of drug use vs no	4.15 (2.55–6.75)	4.06 (2.49–6.61)
LTFU vs cured	MDR-TB vs no	4.58 (2.32–9.03)	4.82 (2.43–9.56)
LTFU vs cured	Underweight vs normal BMI	2.19 (1.27–3.79)	2.21 (1.28–3.83)
LTFU vs cured	Overweight/obesity vs normal BMI	0.91 (0.46–1.81)	0.94 (0.47–1.85)
LTFU vs cured	HIV positive vs negative	1.77 (0.51–6.07)	2.00 (0.56–7.18)
LTFU vs cured	HIV test not performed vs negative	2.51 (1.63–3.88)	2.53 (1.64–3.90)
Death vs cured	Age 22–26 vs ≤21	0.51 (0.13–1.98)	0.53 (0.15–1.92)
Death vs cured	Age 27–37 vs ≤21	0.61 (0.17–2.25)	0.60 (0.17–2.13)
Death vs cured	Age ≥38 vs ≤21	2.39 (0.89–6.40)	2.18 (0.83–5.71)
Death vs cured	Male vs female	1.71 (0.71–4.08)	1.66 (0.72–3.83)
Death vs cured	Incomplete secondary vs complete	2.46 (1.06–5.70)	2.46 (1.10–5.48)
Death vs cured	Weekly alcohol use vs no	0.92 (0.29–2.87)	1.01 (0.34–2.97)
Death vs cured	History of drug use vs no	0.46 (0.12–1.77)	0.47 (0.13–1.73)
Death vs cured	MDR-TB vs no	5.04 (1.62–15.69)	5.69 (1.88–17.19)
Death vs cured	Underweight vs normal BMI	3.77 (1.57–9.03)	3.87 (1.66–9.01)
Death vs cured	Overweight/obesity vs normal BMI	0.27 (0.05–1.55)	0.33 (0.06–1.68)
Death vs cured	HIV positive vs negative	32.12 (8.95–115.33)	35.56 (9.80–129.08)
Death vs cured	HIV test not performed vs negative	3.19 (1.37–7.44)	3.43 (1.53–7.72)
Not evaluated/transferred vs cured	Age 22–26 vs ≤21	1.06 (0.51–2.18)	1.05 (0.51–2.15)
Not evaluated/transferred vs cured	Age 27–37 vs ≤21	1.12 (0.53–2.37)	1.13 (0.54–2.36)
Not evaluated/transferred vs cured	Age ≥38 vs ≤21	0.96 (0.43–2.13)	0.97 (0.44–2.13)
Not evaluated/transferred vs cured	Male vs female	1.32 (0.72–2.44)	1.32 (0.72–2.40)
Not evaluated/transferred vs cured	Incomplete secondary vs complete	1.13 (0.64–2.01)	1.14 (0.66–2.00)
Not evaluated/transferred vs cured	Weekly alcohol use vs no	1.21 (0.59–2.46)	1.24 (0.62–2.48)
Not evaluated/transferred vs cured	History of drug use vs no	1.41 (0.65–3.04)	1.45 (0.69–3.08)
Not evaluated/transferred vs cured	MDR-TB vs no	11.40 (5.86–22.19)	11.69 (6.04–22.63)
Not evaluated/transferred vs cured	Underweight vs normal BMI	0.87 (0.36–2.11)	0.93 (0.39–2.20)
Not evaluated/transferred vs cured	Overweight/obesity vs normal BMI	0.61 (0.24–1.57)	0.65 (0.26–1.62)
Not evaluated/transferred vs cured	HIV positive vs negative	0.24 (0.01–5.90)	0.53 (0.03–10.73)

Not evaluated/transferred vs cured	HIV test not performed vs negative	2.01 (1.08–3.74)	2.06 (1.13–3.77)

Both multinomial models used the same complete-case cohort (n = 1,233), prespecified covariates, and cure as the reference outcome. Ridge estimates used lambda = 0.16 applied to non-intercept coefficients. Mean bias reduction used the Firth/Jeffreys adjusted-likelihood approach. Confidence intervals are Wald 95% intervals. Bias reduction stabilizes estimation under sparse cells but does not eliminate uncertainty caused by the small number of deaths and HIV-positive participants.

Focused binary Firth verification for death versus cure.

**Predictor**	**Firth binary: death vs cure, aOR (95% CI)**
MDR-TB vs no	4.59 (1.44–14.66)
Underweight vs normal BMI	3.63 (1.55–8.49)
HIV positive vs negative	30.71 (8.53–110.49)
HIV test not performed vs negative	3.07 (1.36–6.93)

The focused binary Firth model was restricted to participants who were cured or died and used the same prespecified covariates. It was included as an additional verification of the most sparse and clinically prominent contrast; the multinomial architecture remained the principal analysis.
