# Supplementary material for: How Endpoint Definitions Shape Epidemiologic Inference on Tuberculosis Treatment Outcomes: A Secondary Analysis from Lima, Peru
Source: Infect Dis Rep. 2026 Sep 26;18(5):108. doi: 10.3390/idr18050108 (PMC13704616; doi:10.3390/idr18050108)
Supplement: Supplementary file 1 [file idr-18-00108-s001.zip › Table_S1_STROBE.pdf]

Supplementary Table S1. STROBE checklist for cohort studies.

No.	Recommendation	Location in the manuscript
**TITLE AND ABSTRACT**	**TITLE AND ABSTRACT**	**TITLE AND ABSTRACT**
1	(a) Indicate the study design in the title or abstract using a commonly used term.	Title: "How Endpoint Definitions Shape Epidemiologic Inference on Tuberculosis Treatment Outcomes: A Secondary Analysis from Lima, Peru." Abstract, Methods section: "Secondary analysis of a prospective cohort of adult patients with smear-positive pulmonary tuberculosis."
	(b) Provide an informative and balanced summary in the abstract of what was done and what was found.	The abstract provides an informative and balanced summary of the design, methods, main results, and limitations. It reports the complete-case cohort (n = 1,233), the mutually exclusive outcome counts, the sparse-event numbers (30 deaths, 22 HIV-positive participants, and six HIV-positive deaths), the corrected HIV–death estimate (aOR 32.12; 95% CI 8.95–115.33), and the concordant mean bias-reduced sensitivity estimate (aOR 35.56; 95% CI 9.80–129.08).
**INTRODUCTION**	**INTRODUCTION**	**INTRODUCTION**
2	Background/rationale: Explain the scientific background and rationale for the investigation being reported.	Introduction, opening paragraphs: global, regional, and Peruvian tuberculosis burden and the programmatic relevance of endpoint architecture. A new paragraph explicitly distinguishes the contemporary burden context from the 2010–2011 analytic cohort and notes changes in diagnostic pathways, HIV care, and shorter all-oral MDR/RR-TB regimens.
3	Objectives: State specific objectives, including any prespecified hypotheses.	Introduction, final paragraph: the primary objective is to compare associations with the mutually exclusive outcomes of LTFU, death, and not evaluated/transferred versus documented cure. Composite binary endpoints are explicitly designated as secondary analyses used to evaluate how collapsing categories changes epidemiologic inference.
**METHODS**	**METHODS**	**METHODS**
4	Study design: Present key elements of the design early in the paper.	Methods, subsection "Study design": secondary analysis of a prospective cohort of patients with smear-positive pulmonary tuberculosis treated

		in a high-incidence area of Lima, Peru.<br>Explicit adherence to the STROBE recommendations for observational cohort studies (reference 10).
5	Setting: Describe the setting, locations, and relevant dates, including periods of recruitment, exposure, follow-up, and data collection.	Methods, subsection "Data source": publicly available anonymized secondary data deposited in Dryad (Lima TB Treatment Default Data.xls); original cohort by Lackey et al. (reference 7) with adults enrolled between January 2010 and December 2011 with a first episode of smear-positive pulmonary tuberculosis.<br>Setting: high-incidence area of Lima, Peru. Follow-up of treatment outcomes recorded in health services.
6	(a) Participants: Give the eligibility criteria and the sources and methods of selection, as well as follow-up.	Methods, subsection "Population and sample": patients included in the public Dryad file.<br>Inclusion: records with documented treatment outcome and complete baseline covariates for the complete-case analytic cohort.<br>Exclusion: records without documented outcome and records with missing data in required baseline variables.
	(b) Cohort: For each participant with relevant exposures and outcomes, describe exposure criteria and follow-up methods.	Not applicable beyond the documented treatment outcomes available in the public dataset.
7	Variables: Clearly define all outcomes, exposures, predictors, potential confounders, and effect modifiers. Give diagnostic criteria when applicable.	Methods, subsection "Variables". Primary nominal outcome: documented cure (reference), LTFU, death, and not evaluated/transferred. Transferred out and still in treatment are distinguished conceptually; the latter is a nonterminal status at database closure. Secondary endpoint 1: absence of documented cure excluding ongoing treatment (LTFU, death, or transfer vs cure). Secondary endpoint 2: strict unfavorable outcome (LTFU or death vs cure). The broader closure-status definition that also includes still in treatment is retained only as a sensitivity analysis. Prespecified covariates and reference categories are reported in the same subsection and in Table S2.

8*	Data sources/measurement: For each variable of interest, give sources of data and details of methods of assessment. Describe comparability of assessment methods if there is more than one group.	Methods, subsection "Data source": Lima TB Treatment Default Data.xls file from the Dryad repository (reference 8), including baseline demographic, socioeconomic, behavioral, and clinical variables, treatment outcome, and time to default for patients with that event. Methods, subsection "Variables": terminology updated according to WHO tuberculosis definitions (reference 6); analytic recoding detailed in Table S2; composite-outcome architecture shown in Figure 1.
9	Bias: Describe any efforts to address potential sources of bias.	Methods, subsection "Statistical analysis": prespecified covariate adjustment without automatic selection; ridge-penalized multinomial estimation with lambda = 0.16; approximate Wald heterogeneity tests; unadjusted analyses; complete-case and single stochastic hot-deck missing-data sensitivities; separation of transfer and still-in-treatment components; exclusion of ongoing treatment from the primary composite; extended modeling; standardized marginal probabilities; and a mean bias-reduced multinomial sensitivity analysis for sparse cells. Discussion, subsection "Limitations": residual confounding and limited measurement, sparse outcome–exposure cells, historical external validity, nonterminal outcome definitions, and complete-case uncertainty are explicitly addressed.
10	Study size: Explain how the study size was arrived at.	Methods, subsection "Population and sample" and Results, subsection "Analytic sample". Study size was determined by availability: public file with 1,293 records -> 1,278 with documented outcome -> 1,233 complete-case analytic cohort after excluding records with missing data in required baseline variables. Flow diagram in Figure S1 and missing-data pattern in Table S3.
11	Quantitative variables: Explain how quantitative variables were handled. If applicable, describe which groupings were chosen and why.	Methods, subsection "Variables": age analyzed in the categories available in the dataset; BMI categorized as normal, underweight, and

		overweight/obesity; alcohol use and drug use in prespecified binary categories. Justification: limitations of the original dataset did not allow modeling age, BMI, alcohol use, or drug use on continuous scales. This restriction is explicitly acknowledged in the Discussion, subsection "Limitations".
12	(a) Statistical methods: Describe all statistical methods, including those used to control for confounding.	Methods, subsection "Statistical analysis": baseline-category multinomial logistic regression with cure as the reference. The primary model retained all prespecified covariates and used ridge penalization (lambda = 0.16) only on non-intercept coefficients. Optimization used L-BFGS-B (maxiter = 180; ftol = 1 × 10−10; gtol = 1 × 10−6; maxls = 50), and the model converged after 64 iterations. Wald confidence intervals used the generalized inverse of the penalized Hessian. Heterogeneity was assessed using approximate Wald tests. A mean bias-reduced Firth/Jeffreys multinomial model using the same cohort and covariates was fitted as a sparse-data sensitivity analysis.
	(b) Describe any methods used to examine subgroups and interactions.	Methods, subsection "Statistical analysis": secondary ridge-penalized binary models evaluated absence of documented cure excluding ongoing treatment, strict unfavorable outcome, and the broader closure-status sensitivity definition. The original LTFU contrasts were reproduced. Standardized marginal probabilities were estimated for selected exposure scenarios.
	(c) Explain how missing data were addressed.	Methods, subsections "Population and sample" and "Statistical analysis": the primary analysis used complete cases. Sensitivity analyses used a complete-case denominator restricted to model covariates and a single stochastic hot-deck imputation of covariates among participants with recorded outcomes; the outcome was not imputed. The hot-deck result is explicitly described as a directional sensitivity analysis that does not incorporate between-imputation uncertainty. See Tables S3 and S8.

	(d) If applicable, explain how loss to follow-up was addressed.	Methods, subsection "Statistical analysis" (second paragraph) and Discussion, subsection "Limitations": survival models were not applied because time to event was recorded only for patients with LTFU and comparable censoring or follow-up times were unavailable for cure, death, transfer, or remaining in treatment. Loss to follow-up was modeled as a specific outcome category (LTFU), not as a censoring mechanism.
	(e) Describe any sensitivity analyses.	Methods and Results, subsection "Sensitivity analyses": unadjusted estimates (Table S6); replication of original binary LTFU contrasts (Table S7); missing-data analyses including a single stochastic hot-deck sensitivity (Table S8); component-specific and nonterminal-outcome definitions (Table S9); extended covariate model (Table S10); sparse-data mean bias-reduced analysis and focused binary Firth verification (Table S11); and standardized marginal probabilities (Figure S3).
**RESULTS**	**RESULTS**	**RESULTS**
13*	(a) Participants: Report the numbers of individuals at each stage of the study (potentially eligible, examined, confirmed eligible, included, with complete follow-up, and analyzed).	Results, subsection "Analytic sample". Public file with 1,293 records -> 1,278 with documented outcome -> 1,233 complete-case analytic cohort. Outcome distribution: 1,016 cures, 127 LTFU, 30 deaths, and 60 not evaluated/transferred (40 transferred and 20 still in treatment).
	(b) Give reasons for non-participation at each stage.	Results, subsection "Analytic sample": 15 records lacked a documented outcome and 45 additional records had missing values in baseline covariates required for the primary complete-case model.
	(c) Consider use of a flow diagram.	Participant flow is presented in Figure S1.
14*	(a) Descriptive data: Present characteristics of study participants (demographic, clinical, social) and information on exposures and potential confounders.	Results, subsection "Baseline characteristics" and Table 1. 60.3% male (n = 743); 514 with incomplete secondary education; 233 with weekly alcohol use; 190 with history of drug use; 81 with MDR-TB; 151 underweight; 295 with HIV test not

		performed. Crude covariate distributions in Table S4 and selected exposures in Figure S2.
	(b) Indicate the number of participants with missing data for each variable of interest.	Variable-specific missingness among participants with a recorded outcome is reported in Table S3.
	(c) Summarize follow-up time (e.g., average and total amount).	Comparable follow-up time was unavailable for cure, death, transfer, and still-in-treatment records; this precluded a common time-to-event analysis and is reported in Methods and Limitations.
15*	Outcome data: Report numbers of outcome events or summary measures over time.	Results, subsections "Analytic sample" and "Baseline characteristics". Cure: 1,016 (82.4%); LTFU: 127 (10.3%); death: 30 (2.4%); not evaluated/transferred: 60 (4.9%), composed of 40 transferred patients and 20 patients still in treatment. Detailed distribution by baseline covariates in Table 1 and Table S4.
16	(a) Main results: Present unadjusted estimates and, if applicable, confounder-adjusted estimates and their precision (95% confidence interval). Make clear which confounders were adjusted for and why they were included.	Results, subsection “Primary multinomial model”, Figure 2, and Table S5. LTFU vs cure: drug use aOR 4.15 (95% CI 2.55–6.75), MDR-TB 4.58 (2.32–9.03), HIV testing not performed 2.51 (1.63–3.88), underweight 2.19 (1.27–3.79), weekly alcohol use 2.12 (1.32–3.41), and incomplete secondary education 1.77 (1.17–2.69). Death vs cure: HIV-positive status 32.12 (8.95–115.33), based on six deaths among 22 HIV-positive participants; MDR-TB 5.04 (1.62–15.69); underweight 3.77 (1.57–9.03). Not evaluated/transferred vs cure: MDR-TB 11.40 (5.86–22.19). The mean bias-reduced estimates are reported in Table S11.
	(b) Report category boundaries when continuous variables were categorized.	
	(c) If relevant, consider translating estimates of relative risk into absolute risk for a meaningful time period.	Results, subsection "Sensitivity analyses" and Figure S3. Standardized marginal probabilities: drug use with LTFU probability of 22.0% (exposed) versus 7.0% (reference); MDR-TB with cure probability of 51.2% versus 84.5% and not evaluated/transferred probability of 23.1% versus 3.7%; HIV-positive status with

		death probability of 24.2% versus 2.0%.
17	Other analyses: Report other analyses performed (e.g., subgroup, interaction, and sensitivity analyses).	Results, subsections "Composite outcomes" and "Sensitivity analyses". The revised primary composite excludes ongoing treatment: absence of documented cure excluding ongoing treatment vs cure yielded aORs of 4.40 for MDR-TB, 3.93 for HIV-positive status, 2.76 for drug use, 2.61 for HIV testing not performed, 2.04 for underweight, 1.78 for weekly alcohol use, and 1.60 for incomplete secondary education (Table 2 and Table S9). Strict unfavorable-outcome results are also reported. Tables S6–S11 and Figure S3 present the unadjusted, replication, missing-data, nonterminal, extended, sparse-data, and absolute-probability analyses.
**DISCUSSION**	**DISCUSSION**	**DISCUSSION**
18	Key results: Summarize key findings in relation to the study objectives.	Discussion, subsection "Main Findings": associations differed across mutually exclusive categories; the HIV–death estimate was large but imprecise; the combined MDR-TB association with not evaluated/transferred was largely driven by participants still in treatment; and composite odds ratios were interpreted as mixtures of component-specific processes rather than estimates of a single mechanism.
19	Limitations: Discuss study limitations, considering potential sources of bias or imprecision. Discuss the direction and magnitude of any potential bias.	Discussion, subsection "Limitations": historical cohort and changed contemporary treatment context; absence of causal identification; coarse covariate measurement; 30 deaths, 22 HIV-positive participants, and six HIV-positive deaths; model sensitivity under sparse cells despite penalization; conceptual nonequivalence of transfer and still in treatment; and uncertainty under complete-case and single hot-deck analyses. The likely direction is increased imprecision and model dependence rather than resolution of sparse information by aggregation.
20	Interpretation: Give a cautious overall interpretation of results considering objectives, limitations, multiplicity of analyses, results from similar studies, and other relevant evidence.	Discussion: the interpretation is explicitly noncausal and recognizes that associations reported across studies are not uniform and are difficult to compare because of differing endpoint

		definitions, treatment regimens, follow-up windows, and adjustment sets. Composite endpoints are described as clinically heterogeneous. Results from the mean bias-reduced model support the direction of the primary findings but not precise estimation of the HIV–death magnitude.
21	Generalizability: Discuss the generalizability (external validity) of the study results.	Introduction, Discussion, Limitations, and Conclusions: the cohort reflects care in 2010–2011, whereas current burden estimates provide programmatic context. Changes in diagnostics, HIV care, and shorter all-oral MDR/RR-TB regimens limit transportability. The findings are presented primarily as a methodological demonstration of endpoint sensitivity rather than contemporary risk estimates for Lima.
**OTHER INFORMATION**	**OTHER INFORMATION**	**OTHER INFORMATION**
22	Funding: Give the source of funding and the role of the funders for the present study and, if applicable, for the original study on which the present article is based.	Funding statement: article-processing charges, if applicable, will be covered by the Vicerrectorado de Investigación of UNTRM, which had no role in the study. Ethics statements: the original cohort was approved by the Institutional Review Board of Universidad Peruana Cayetano Heredia and the East Lima DISA, and participants provided written informed consent. The present analysis used publicly available anonymized data without access to direct identifiers or participant contact. Data are available from Dryad at https://datadryad.org/dataset/doi:10.5061/dryad.fp94d.

STROBE = Strengthening the Reporting of Observational Studies in Epidemiology.
