# Supplementary material for: How Endpoint Definitions Shape Epidemiologic Inference on Tuberculosis Treatment Outcomes: A Secondary Analysis from Lima, Peru
Source: Infect Dis Rep. 2026 Sep 26;18(5):108. doi: 10.3390/idr18050108 (PMC13704616; doi:10.3390/idr18050108)
Supplement: Supplementary file 1 [file idr-18-00108-s001.zip › Table_S2_Dictionary.pdf]

Supplementary Table S2. Variable dictionary and analytic recoding.

Original/derived variable	Type	Original categories	Analytic recoding	Reference category	Comment
Treatment outcome	Categorical outcome	Cured; Default; Died; Transferred; Still in treatment	Cured; LTFU; Died; Not evaluated/transferred	Cured	"Default" was relabeled as LTFU. "Transferred" and "Still in treatment" were combined only in the primary nominal category representing absence of a documented final outcome at the source facility; transferred out denotes an unknown final outcome after transfer, whereas still in treatment is a nonterminal status at database closure. Components were examined separately in sensitivity analyses.
Age	Categorical predictor	21 years or younger; 22 to 26; 27 to 37; 38 or older	Indicator variables by age group	<=21 years	Age was not available as a continuous variable.
Sex	Binary predictor	Female; Male	Male vs female	Female	Demographic covariate forced into the model.
Completed secondary education	Binary predictor	Yes; No	Incomplete secondary education vs complete	Yes	Marker of social vulnerability.
Alcohol use at least once per week	Binary predictor	No; Yes	Weekly alcohol use vs no	No	Behavioral exposure.
History of drug use	Binary predictor	No; Yes	Any history of drug use vs no	No	Behavioral exposure.
MDR-TB	Binary predictor	No; Yes	MDR-TB vs no	No	Clinical marker of resistance.
Body mass index	Categorical predictor	Normal; Underweight; Overweight/obesity	Indicators for underweight and overweight/obesity	Normal	BMI was not available as a continuous variable.

HIV status	Categorical predictor	Negative; Positive; Test not performed	Indicators for positive and test not performed	Negative	"Test not performed" was treated as an observed programmatic condition, not as missing data.
absence_documented_cure_excluding_ongoing	Derived binary outcome	Derived	LTFU + died + transferred vs cured; still in treatment excluded	Cured	Primary composite analysis of absence of documented cure that excludes ongoing treatment because it is nonterminal.
closure_status_absence_of_cure	Derived binary outcome	Derived	LTFU + died + transferred + still in treatment vs cured	Cured	Broader closure-status sensitivity definition; not interpreted as a clinically homogeneous unfavorable outcome.
strict_unfavorable_outcome	Derived binary outcome	Derived	LTFU + death vs cured; transferred/still in treatment excluded	Cured	Strict unfavorable outcome that excludes non-evaluable outcomes.
