# Supplementary material for: How Endpoint Definitions Shape Epidemiologic Inference on Tuberculosis Treatment Outcomes: A Secondary Analysis from Lima, Peru
Source: Infect Dis Rep. 2026 Sep 26;18(5):108. doi: 10.3390/idr18050108 (PMC13704616; doi:10.3390/idr18050108)
Supplement: Supplementary file 1 [file idr-18-00108-s001.zip › Table_S3_Missing.pdf]

Supplementary Table S3. Missing baseline data among patients with recorded outcome.

Variable	Missing, total n=1,278	Missing among cured	Missing among LTFU	Missing among died	Missing among not evaluated/transferred
Age	0	0	0	0	0
Sex	0	0	0	0	0
Marital status	0	0	0	0	0
Poverty status	23	13	9	0	1
History of imprisonment	2	2	0	0	0
Completed secondary education	1	1	0	0	0
History of tobacco use	3	3	0	0	0
Weekly alcohol use	10	8	1	0	1
History of drug use	0	0	0	0	0
History of rehabilitation	3	3	0	0	0
MDR-TB	0	0	0	0	0
Body mass index	12	8	3	0	1
History of chronic disease	0	0	0	0	0
HIV status	0	0	0	0	0
History of diabetes mellitus	1	1	0	0	0
