# Supplementary material for: How Endpoint Definitions Shape Epidemiologic Inference on Tuberculosis Treatment Outcomes: A Secondary Analysis from Lima, Peru
Source: Infect Dis Rep. 2026 Sep 26;18(5):108. doi: 10.3390/idr18050108 (PMC13704616; doi:10.3390/idr18050108)
Supplement: Supplementary file 1 [file idr-18-00108-s001.zip › Table_S4_Crude.pdf]

Supplementary Table S4. Crude outcome distribution by covariate level.

**Variable**	**Category**	**n**	**Cured**	**LTFU**	**Died**	**Not evaluated/transferred**	**Closure-status no cure (sensitivity)**
Age group	21 or younger	321	263 (81.9)	37 (11.5)	6 (1.9)	15 (4.7)	58 (18.1)
Age group	22 to 26	326	270 (82.8)	36 (11.0)	3 (0.9)	17 (5.2)	56 (17.2)
Age group	27 to 37	291	233 (80.1)	39 (13.4)	4 (1.4)	15 (5.2)	58 (19.9)
Age group	38 or older	295	250 (84.7)	15 (5.1)	17 (5.8)	13 (4.4)	45 (15.3)
Sex	Female	490	433 (88.4)	29 (5.9)	9 (1.8)	19 (3.9)	57 (11.6)
Sex	Male	743	583 (78.5)	98 (13.2)	21 (2.8)	41 (5.5)	160 (21.5)
Marital status	Single	637	525 (82.4)	70 (11.0)	11 (1.7)	31 (4.9)	112 (17.6)
Marital status	Married or cohabiting	469	391 (83.4)	45 (9.6)	9 (1.9)	24 (5.1)	78 (16.6)
Marital status	Divorced or separated	91	72 (79.1)	10 (11.0)	5 (5.5)	4 (4.4)	19 (20.9)
Marital status	Widowed	36	28 (77.8)	2 (5.6)	5 (13.9)	1 (2.8)	8 (22.2)
Poverty status	No poverty	1,024	852 (83.2)	97 (9.5)	24 (2.3)	51 (5.0)	172 (16.8)
Poverty status	Poverty/extreme poverty	209	164 (78.5)	30 (14.4)	6 (2.9)	9 (4.3)	45 (21.5)
History of imprisonment	No	1,178	984 (83.5)	109 (9.3)	29 (2.5)	56 (4.8)	194 (16.5)
History of imprisonment	Yes	55	32 (58.2)	18 (32.7)	1 (1.8)	4 (7.3)	23 (41.8)
Completed secondary education	Yes	719	622 (86.5)	52 (7.2)	10 (1.4)	35 (4.9)	97 (13.5)
Completed secondary education	No	514	394 (76.7)	75 (14.6)	20 (3.9)	25 (4.9)	120 (23.3)
Tobacco use	Never smoked	742	638 (86.0)	50 (6.7)	18 (2.4)	36 (4.9)	104 (14.0)
Tobacco use	Former smoker	440	337 (76.6)	69 (15.7)	12 (2.7)	22 (5.0)	103 (23.4)
Tobacco use	Current smoker	51	41 (80.4)	8 (15.7)	0 (0.0)	2 (3.9)	10 (19.6)
Weekly alcohol use	No	1,000	855 (85.5)	72 (7.2)	26 (2.6)	47 (4.7)	145 (14.5)
Weekly alcohol use	Yes	233	161 (69.1)	55 (23.6)	4 (1.7)	13 (5.6)	72 (30.9)
History of drug use	No	1,043	903 (86.6)	64 (6.1)	27 (2.6)	49 (4.7)	140 (13.4)
History of drug use	Yes	190	113 (59.5)	63 (33.2)	3 (1.6)	11 (5.8)	77 (40.5)

History of rehabilitation	No	1,156	970 (83.9)	102 (8.8)	29 (2.5)	55 (4.8)	186 (16.1)
History of rehabilitation	Yes	77	46 (59.7)	25 (32.5)	1 (1.3)	5 (6.5)	31 (40.3)
MDR-TB	No	1,152	974 (84.5)	111 (9.6)	25 (2.2)	42 (3.6)	178 (15.5)
MDR-TB	Yes	81	42 (51.9)	16 (19.8)	5 (6.2)	18 (22.2)	39 (48.1)
BMI	Normal	916	757 (82.6)	91 (9.9)	19 (2.1)	49 (5.3)	159 (17.4)
BMI	Underweight	151	110 (72.8)	25 (16.6)	10 (6.6)	6 (4.0)	41 (27.2)
BMI	Overweight/obesity	166	149 (89.8)	11 (6.6)	1 (0.6)	5 (3.0)	17 (10.2)
Chronic disease	No	1,198	986 (82.3)	125 (10.4)	28 (2.3)	59 (4.9)	212 (17.7)
Chronic disease	Yes	35	30 (85.7)	2 (5.7)	2 (5.7)	1 (2.9)	5 (14.3)
HIV status	Negative	916	788 (86.0)	72 (7.9)	13 (1.4)	43 (4.7)	128 (14.0)
HIV status	Positive	22	12 (54.5)	4 (18.2)	6 (27.3)	0 (0.0)	10 (45.5)
HIV status	Test not performed	295	216 (73.2)	51 (17.3)	11 (3.7)	17 (5.8)	79 (26.8)
History of diabetes mellitus	No	1,179	969 (82.2)	127 (10.8)	28 (2.4)	55 (4.7)	210 (17.8)
History of diabetes mellitus	Yes	54	47 (87.0)	0 (0.0)	2 (3.7)	5 (9.3)	7 (13.0)

The closure-status sensitivity column combines LTFU, death, transferred out, and still in treatment. In the revised manuscript, the primary composite excludes participants still in treatment because this is a nonterminal status; the broader definition is retained only as a sensitivity analysis.
