# Supplementary material for: How Endpoint Definitions Shape Epidemiologic Inference on Tuberculosis Treatment Outcomes: A Secondary Analysis from Lima, Peru
Source: Infect Dis Rep. 2026 Sep 26;18(5):108. doi: 10.3390/idr18050108 (PMC13704616; doi:10.3390/idr18050108)
Supplement: Supplementary file 1 [file idr-18-00108-s001.zip › Table_S5_Multinomial.pdf]

Supplementary Table S5. Primary ridge-penalized multinomial model—complete adjusted estimates.

Outcome contrast	Predictor	aOR (95% CI)	p	Approximate p for heterogeneity
LTFU vs Cured	Age 22-26 vs <=21	0.83 (0.48–1.41)	0.483	0.625
LTFU vs Cured	Age 27-37 vs <=21	1.11 (0.65–1.88)	0.708	0.688
LTFU vs Cured	Age >=38 vs <=21	0.50 (0.26–0.98)	0.043	0.026
LTFU vs Cured	Male vs female	1.18 (0.71–1.96)	0.530	0.757
LTFU vs Cured	Incomplete secondary education vs complete	1.77 (1.17–2.69)	0.007	0.243
LTFU vs Cured	Weekly alcohol use vs no	2.12 (1.32–3.41)	0.002	0.203
LTFU vs Cured	History of drug use vs no	4.15 (2.55–6.75)	<0.001	<0.001
LTFU vs Cured	MDR-TB vs no	4.58 (2.32–9.03)	<0.001	0.084
LTFU vs Cured	Underweight vs normal BMI	2.19 (1.27–3.79)	0.005	0.050
LTFU vs Cured	Overweight/obesity vs normal BMI	0.91 (0.46–1.81)	0.788	0.400
LTFU vs Cured	HIV positive vs negative	1.77 (0.51–6.07)	0.367	<0.001
LTFU vs Cured	HIV test not performed vs negative	2.51 (1.63–3.88)	<0.001	0.662
Died vs Cured	Age 22-26 vs <=21	0.51 (0.13–1.98)	0.330	0.625
Died vs Cured	Age 27-37 vs <=21	0.61 (0.17–2.25)	0.461	0.688
Died vs Cured	Age >=38 vs <=21	2.39 (0.89–6.40)	0.084	0.026
Died vs Cured	Male vs female	1.71 (0.71–4.08)	0.228	0.757
Died vs Cured	Incomplete secondary education vs complete	2.46 (1.06–5.70)	0.036	0.243
Died vs Cured	Weekly alcohol use vs no	0.92 (0.29–2.87)	0.880	0.203
Died vs Cured	History of drug use vs no	0.46 (0.12–1.77)	0.257	<0.001
Died vs Cured	MDR-TB vs no	5.04 (1.62–15.69)	0.005	0.084
Died vs Cured	Underweight vs normal BMI	3.77 (1.57–9.03)	0.003	0.050
Died vs Cured	Overweight/obesity vs normal BMI	0.27 (0.05–1.55)	0.142	0.400
Died vs Cured	HIV positive vs negative	32.12 (8.95–115.33)	<0.001	<0.001
Died vs Cured	HIV test not performed vs negative	3.19 (1.37–7.44)	0.007	0.662
Not evaluated/transferred vs Cured	Age 22-26 vs <=21	1.06 (0.51–2.18)	0.883	0.625
Not evaluated/transferred vs Cured	Age 27-37 vs <=21	1.12 (0.53–2.37)	0.769	0.688
Not evaluated/transferred vs Cured	Age >=38 vs <=21	0.96 (0.43–2.13)	0.922	0.026

Not evaluated/transferred vs Cured	Male vs female	1.32 (0.72–2.44)	0.371	0.757
Not evaluated/transferred vs Cured	Incomplete secondary education vs complete	1.13 (0.64–2.01)	0.665	0.243
Not evaluated/transferred vs Cured	Weekly alcohol use vs no	1.21 (0.59–2.46)	0.605	0.203
Not evaluated/transferred vs Cured	History of drug use vs no	1.41 (0.65–3.04)	0.387	<0.001
Not evaluated/transferred vs Cured	MDR-TB vs no	11.40 (5.86–22.19)	<0.001	0.084
Not evaluated/transferred vs Cured	Underweight vs normal BMI	0.87 (0.36–2.11)	0.752	0.050
Not evaluated/transferred vs Cured	Overweight/obesity vs normal BMI	0.61 (0.24–1.57)	0.307	0.400
Not evaluated/transferred vs Cured	HIV positive vs negative	0.24 (0.01–5.90)	0.384	<0.001
Not evaluated/transferred vs Cured	HIV test not performed vs negative	2.01 (1.08–3.74)	0.027	0.662

Adjusted odds ratios (aORs) with 95% confidence intervals were estimated using baseline-category multinomial logistic regression with "Cured" as the reference outcome. The model included all prespecified covariates. Ridge penalization (lambda = 0.16) was applied only to non-intercept coefficients. Wald confidence intervals were calculated from the generalized inverse of the penalized Hessian. Heterogeneity p values are approximate Wald tests of equality of coefficients across LTFU, death, and not evaluated/transferred categories.
