# Supplementary material for: How Endpoint Definitions Shape Epidemiologic Inference on Tuberculosis Treatment Outcomes: A Secondary Analysis from Lima, Peru
Source: Infect Dis Rep. 2026 Sep 26;18(5):108. doi: 10.3390/idr18050108 (PMC13704616; doi:10.3390/idr18050108)
Supplement: Supplementary file 1 [file idr-18-00108-s001.zip › Table_S6_Unadjusted.pdf]

Supplementary Table S6. Unadjusted outcome-specific associations.

Outcome contrast	Predictor	aOR (95% CI)	p
LTFU vs Cured	Age 22-26 vs <=21	1.09 (0.73–1.65)	0.669
Died vs Cured	Age 22-26 vs <=21	0.33 (0.10–1.02)	0.055
Not evaluated/transferred vs Cured	Age 22-26 vs <=21	1.09 (0.61–1.94)	0.764
LTFU vs Cured	Age 27-37 vs <=21	1.49 (0.99–2.22)	0.054
Died vs Cured	Age 27-37 vs <=21	0.53 (0.19–1.49)	0.229
Not evaluated/transferred vs Cured	Age 27-37 vs <=21	1.12 (0.62–2.03)	0.714
LTFU vs Cured	Age >=38 vs <=21	0.41 (0.24–0.72)	0.002
Died vs Cured	Age >=38 vs <=21	3.89 (1.88–8.04)	<0.001
Not evaluated/transferred vs Cured	Age >=38 vs <=21	0.85 (0.45–1.59)	0.609
LTFU vs Cured	Male vs female	2.49 (1.62–3.83)	<0.001
Died vs Cured	Male vs female	1.71 (0.78–3.72)	0.178
Not evaluated/transferred vs Cured	Male vs female	1.59 (0.91–2.77)	0.100
LTFU vs Cured	Incomplete secondary education vs complete	2.26 (1.56–3.29)	<0.001
Died vs Cured	Incomplete secondary education vs complete	3.07 (1.44–6.54)	0.004
Not evaluated/transferred vs Cured	Incomplete secondary education vs complete	1.12 (0.66–1.90)	0.661
LTFU vs Cured	Weekly alcohol use vs no	4.02 (2.73–5.93)	<0.001
Died vs Cured	Weekly alcohol use vs no	0.82 (0.29–2.32)	0.713
Not evaluated/transferred vs Cured	Weekly alcohol use vs no	1.46 (0.77–2.74)	0.243
LTFU vs Cured	History of drug use vs no	7.75 (5.21–11.54)	<0.001
Died vs Cured	History of drug use vs no	0.89 (0.28–2.87)	0.846
Not evaluated/transferred vs Cured	History of drug use vs no	1.77 (0.90–3.48)	0.099
LTFU vs Cured	MDR-TB vs no	3.24 (1.77–5.92)	<0.001
Died vs Cured	MDR-TB vs no	4.30 (1.58–11.72)	0.004
Not evaluated/transferred vs Cured	MDR-TB vs no	9.47 (5.05–17.76)	<0.001
LTFU vs Cured	Underweight vs normal BMI	2.00 (1.24–3.23)	0.004
Died vs Cured	Underweight vs normal BMI	3.97 (1.82–8.65)	<0.001
Not evaluated/transferred vs Cured	Underweight vs normal BMI	0.91 (0.39–2.14)	0.837
LTFU vs Cured	Overweight/obesity vs normal BMI	0.56 (0.30–1.05)	0.072
Died vs Cured	Overweight/obesity vs normal BMI	0.25 (0.05–1.36)	0.109
Not evaluated/transferred vs Cured	Overweight/obesity vs normal BMI	0.54 (0.22–1.34)	0.186
LTFU vs Cured	HIV positive vs negative	2.53 (0.82–7.79)	0.106
Died vs Cured	HIV positive vs negative	18.30 (6.40–52.35)	<0.001

Not evaluated/transferred vs Cured	HIV positive vs negative	0.28 (0.01–7.24)	0.441
LTFU vs Cured	HIV test not performed vs negative	2.47 (1.68–3.63)	<0.001
Died vs Cured	HIV test not performed vs negative	2.10 (0.99–4.46)	0.052
Not evaluated/transferred vs Cured	HIV test not performed vs negative	1.45 (0.82–2.59)	0.204
