# Supplementary material for: How Endpoint Definitions Shape Epidemiologic Inference on Tuberculosis Treatment Outcomes: A Secondary Analysis from Lima, Peru
Source: Infect Dis Rep. 2026 Sep 26;18(5):108. doi: 10.3390/idr18050108 (PMC13704616; doi:10.3390/idr18050108)
Supplement: Supplementary file 1 [file idr-18-00108-s001.zip › Table_S7_Replication.pdf]

Supplementary Table S7. Replication of the original contrasts for LTFU.

**Predictor**	**LTFU vs all other outcomes, aOR (95% CI)**	**LTFU vs all others, p**	**LTFU vs cured only, aOR (95% CI)**	**LTFU vs cured only, p**
Age 22-26 vs <=21	0.83 (0.49–1.41)	0.497	0.79 (0.46–1.36)	0.395
Age 27-37 vs <=21	1.10 (0.65–1.86)	0.718	1.09 (0.64–1.87)	0.744
Age >=38 vs <=21	0.47 (0.24–0.91)	0.026	0.48 (0.24–0.93)	0.030
Weekly alcohol use vs no	2.10 (1.31–3.35)	0.002	2.02 (1.25–3.26)	0.004
HIV positive vs negative	1.38 (0.42–4.50)	0.595	1.55 (0.44–5.48)	0.500
HIV test not performed vs negative	2.31 (1.50–3.55)	<0.001	2.47 (1.59–3.83)	<0.001
History of drug use vs no	4.13 (2.56–6.66)	<0.001	4.00 (2.45–6.53)	<0.001
Incomplete secondary education vs complete	1.70 (1.12–2.58)	0.012	1.81 (1.19–2.77)	0.006
MDR-TB vs no	2.97 (1.54–5.71)	0.001	4.36 (2.16–8.82)	<0.001
Male vs female	1.13 (0.68–1.87)	0.641	1.26 (0.76–2.10)	0.377
Overweight/obesity vs normal BMI	0.97 (0.49–1.92)	0.933	0.92 (0.46–1.83)	0.815
Underweight vs normal BMI	2.09 (1.22–3.58)	0.008	2.41 (1.38–4.19)	0.002

To assess concordance with the binary formulation of the original study, two specifications were reproduced: (1) LTFU versus all other aggregated outcomes and (2) LTFU versus cure only. The direction and magnitude of the associations were consistent with the original publication.
