# Supplementary material for: How Endpoint Definitions Shape Epidemiologic Inference on Tuberculosis Treatment Outcomes: A Secondary Analysis from Lima, Peru
Source: Infect Dis Rep. 2026 Sep 26;18(5):108. doi: 10.3390/idr18050108 (PMC13704616; doi:10.3390/idr18050108)
Supplement: Supplementary file 1 [file idr-18-00108-s001.zip › Table_S8_MissingSensitivity.pdf]

Supplementary Table S8. Missing-data sensitivity analysis, including a single stochastic hot-deck imputation.

Outcome contrast	Predictor	Primary complete-case, n=1,233 aOR (95% CI)	Complete-case for model covariates, n=1,255 aOR (95% CI)	Single stochastic hot-deck, n=1,278 aOR (95% CI)
LTFU vs Cured	Age 22-26 vs <=21	0.83 (0.48–1.41)	0.89 (0.52–1.50)	0.83 (0.50–1.40)
LTFU vs Cured	Age 27-37 vs <=21	1.11 (0.65–1.88)	1.18 (0.70–1.99)	1.13 (0.67–1.90)
LTFU vs Cured	Age >=38 vs <=21	0.50 (0.26–0.98)	0.53 (0.28–1.02)	0.51 (0.27–0.97)
LTFU vs Cured	Male vs female	1.18 (0.71–1.96)	1.19 (0.72–1.99)	1.19 (0.72–1.98)
LTFU vs Cured	Incomplete secondary education vs complete	1.77 (1.17–2.69)	1.75 (1.16–2.64)	1.75 (1.16–2.63)
LTFU vs Cured	Weekly alcohol use vs no	2.12 (1.32–3.41)	2.11 (1.32–3.35)	2.25 (1.42–3.54)
LTFU vs Cured	History of drug use vs no	4.15 (2.55–6.75)	4.42 (2.75–7.11)	4.39 (2.75–7.00)
LTFU vs Cured	MDR-TB vs no	4.58 (2.32–9.03)	4.70 (2.40–9.21)	4.29 (2.20–8.35)
LTFU vs Cured	Underweight vs normal BMI	2.19 (1.27–3.79)	2.28 (1.34–3.91)	2.21 (1.30–3.78)
LTFU vs Cured	Overweight/obesity vs normal BMI	0.91 (0.46–1.81)	0.89 (0.45–1.78)	0.87 (0.44–1.73)
LTFU vs Cured	HIV positive vs negative	1.77 (0.51–6.07)	2.13 (0.67–6.77)	2.14 (0.67–6.76)
LTFU vs Cured	HIV test not performed vs negative	2.51 (1.63–3.88)	2.65 (1.73–4.06)	2.57 (1.69–3.92)
Died vs Cured	Age 22-26 vs <=21	0.51 (0.13–1.98)	0.51 (0.13–1.97)	0.50 (0.13–1.94)
Died vs Cured	Age 27-37 vs <=21	0.61 (0.17–2.25)	0.60 (0.16–2.22)	0.59 (0.16–2.18)
Died vs Cured	Age >=38 vs <=21	2.39 (0.89–6.40)	2.37 (0.89–6.36)	2.37 (0.88–6.36)
Died vs Cured	Male vs female	1.71 (0.71–4.08)	1.70 (0.71–4.07)	1.68 (0.70–4.03)
Died vs Cured	Incomplete secondary education vs complete	2.46 (1.06–5.70)	2.47 (1.07–5.73)	2.48 (1.07–5.75)
Died vs Cured	Weekly alcohol use vs no	0.92 (0.29–2.87)	0.93 (0.30–2.89)	0.95 (0.30–2.96)
Died vs Cured	History of drug use vs no	0.46 (0.12–1.77)	0.44 (0.12–1.67)	0.43 (0.11–1.63)
Died vs Cured	MDR-TB vs no	5.04 (1.62–15.69)	5.20 (1.67–16.17)	5.08 (1.64–15.76)
Died vs Cured	Underweight vs normal BMI	3.77 (1.57–9.03)	3.81 (1.59–9.13)	3.80 (1.58–9.11)
Died vs Cured	Overweight/obesity vs normal BMI	0.27 (0.05–1.55)	0.27 (0.05–1.56)	0.27 (0.05–1.55)
Died vs Cured	HIV positive vs negative	32.12 (8.95–115.33)	33.77 (9.40–121.29)	34.41 (9.58–123.56)
Died vs Cured	HIV test not performed vs negative	3.19 (1.37–7.44)	3.26 (1.40–7.60)	3.25 (1.39–7.57)
Not evaluated/transferred vs Cured	Age 22-26 vs <=21	1.06 (0.51–2.18)	0.99 (0.48–2.02)	1.06 (0.52–2.13)
Not evaluated/transferred vs Cured	Age 27-37 vs <=21	1.12 (0.53–2.37)	1.05 (0.50–2.20)	1.02 (0.49–2.14)

Not evaluated/transferred vs Cured	Age >=38 vs <=21	0.96 (0.43–2.13)	0.89 (0.40–1.95)	0.87 (0.39–1.90)
Not evaluated/transferred vs Cured	Male vs female	1.32 (0.72–2.44)	1.34 (0.72–2.46)	1.34 (0.73–2.46)
Not evaluated/transferred vs Cured	Incomplete secondary education vs complete	1.13 (0.64–2.01)	1.18 (0.67–2.07)	1.25 (0.72–2.18)
Not evaluated/transferred vs Cured	Weekly alcohol use vs no	1.21 (0.59–2.46)	1.16 (0.57–2.34)	1.21 (0.61–2.41)
Not evaluated/transferred vs Cured	History of drug use vs no	1.41 (0.65–3.04)	1.48 (0.70–3.12)	1.49 (0.72–3.09)
Not evaluated/transferred vs Cured	MDR-TB vs no	11.40 (5.86–22.19)	11.27 (5.81–21.88)	11.05 (5.79–21.08)
Not evaluated/transferred vs Cured	Underweight vs normal BMI	0.87 (0.36–2.11)	0.84 (0.34–2.03)	0.82 (0.34–1.99)
Not evaluated/transferred vs Cured	Overweight/obesity vs normal BMI	0.61 (0.24–1.57)	0.61 (0.24–1.55)	0.70 (0.29–1.69)
Not evaluated/transferred vs Cured	HIV positive vs negative	0.24 (0.01–5.90)	0.24 (0.01–5.93)	0.24 (0.01–5.75)
Not evaluated/transferred vs Cured	HIV test not performed vs negative	2.01 (1.08–3.74)	1.98 (1.07–3.66)	1.85 (1.01–3.41)

Comparison of aORs across three missing-data handling strategies: the primary complete-case analysis, a complete-case analysis restricted to model covariates, and a single stochastic hot-deck imputation among participants with a recorded outcome. The outcome was not imputed. The single hot-deck analysis does not incorporate between-imputation uncertainty and should be interpreted only as a directional sensitivity analysis, not as a full multiple-imputation correction.
