# Supplementary material for: How Endpoint Definitions Shape Epidemiologic Inference on Tuberculosis Treatment Outcomes: A Secondary Analysis from Lima, Peru
Source: Infect Dis Rep. 2026 Sep 26;18(5):108. doi: 10.3390/idr18050108 (PMC13704616; doi:10.3390/idr18050108)
Supplement: Supplementary file 1 [file idr-18-00108-s001.zip › Table_S9_NonterminalSensitivity.pdf]

Supplementary Table S9. Sensitivity analysis for transferred and still-in-treatment definitions.

Predictor	Absence of documented cure, excluding ongoing treatment, vs cured	Not evaluated/transferred vs cured	Still in treatment vs cured	Transferred vs cured
Age 22-26 vs <=21	0.83 (0.52–1.32)	1.04 (0.50–2.16)	1.12 (0.34–3.71)	0.99 (0.40–2.44)
Age 27-37 vs <=21	1.10 (0.69–1.74)	1.03 (0.48–2.20)	0.72 (0.20–2.64)	1.23 (0.50–3.04)
Age >=38 vs <=21	0.95 (0.59–1.55)	0.91 (0.41–2.04)	0.30 (0.06–1.50)	1.38 (0.55–3.46)
Weekly alcohol use vs no	1.78 (1.18–2.68)	1.21 (0.59–2.49)	0.43 (0.09–2.06)	1.52 (0.69–3.36)
HIV positive vs negative	3.93 (1.54–10.06)	0.18 (0.01–3.94)	0.36 (0.01–12.55)	0.30 (0.01–8.72)
HIV test not performed vs negative	2.61 (1.82–3.74)	1.92 (1.03–3.57)	0.77 (0.17–3.55)	2.26 (1.15–4.43)
History of drug use vs no	2.76 (1.81–4.22)	1.47 (0.68–3.19)	1.39 (0.31–6.24)	1.43 (0.59–3.46)
Incomplete secondary education vs complete	1.60 (1.13–2.26)	1.12 (0.62–2.01)	2.17 (0.75–6.27)	0.86 (0.43–1.72)
MDR-TB vs no	4.40 (2.45–7.88)	11.74 (6.01–22.94)	49.92 (18.06–138.01)	3.17 (1.04–9.66)
Male vs female	1.36 (0.92–2.03)	1.33 (0.72–2.48)	1.00 (0.34–2.90)	1.54 (0.72–3.27)
Overweight/obesity vs normal BMI	0.69 (0.39–1.22)	0.59 (0.23–1.52)	0.30 (0.04–1.99)	0.65 (0.23–1.85)
Underweight vs normal BMI	2.04 (1.29–3.22)	0.84 (0.34–2.10)	1.46 (0.40–5.27)	0.53 (0.13–2.07)

Alternative definitions clarify the role of nonterminal records: (i) absence of documented cure excluding ongoing treatment (LTFU, death, or transfer vs cure); (ii) the combined not evaluated/transferred nominal category; (iii) still in treatment alone; and (iv) transfer alone. Still in treatment is a nonterminal status at database closure and is excluded from the revised primary composite. The combined nominal category and the broader closure-status definition are not interpreted as clinically homogeneous treatment failure.
